# The Impact of Implementing Indicators of Quality of Oncological Care on Improving Patient Outcomes: A Cross-Sectional Review of Experiences from Countries Using Indicators in the Quality Assessment Process

**DOI:** 10.3390/cancers17081362

**Published:** 2025-04-18

**Authors:** Karolina Piekarska, Piotr Bednarski, Barbara Politynska, Anna M. Wojtukiewicz, Maciej Krzakowski, Marek Z. Wojtukiewicz

**Affiliations:** 1 Department of Oncology, Medical University of Bialystok, 15-027 Bialystok, Poland; k.piekarska.85@gmail.com (K.P.); bednarski.nsk@gmail.com (P.B.); 2 Department of Psychology and Philosophy, Medical University of Bialystok, 15-295 Bialystok, Polandaniawojtukiewicz@gmail.com (A.M.W.); 3 National Cancer Institute, 02-781 Warsaw, Poland; maciej.krzakowski@pib-nio.pl

**Keywords:** quality indicators, patient outcomes, survival rates, cancer plans, cancer management

## Abstract

The implementation of QIs in the pursuit of improving patient outcomes in oncological care has become a primary goal for many countries. The purpose of this cross-sectional review is to present the experiences of several countries that have implemented different strategies in using QIs to assess the quality of cancer care. Countries such as the United States, the United Kingdom, Canada, the Netherlands, Australia, and Israel have been pioneers in integrating QIs into their healthcare systems, which has led to significant improvements in the delivery of care. These indicators help assess adherence to clinical guidelines, timeliness of treatment, safety of practices, and overall patient survival. Data from these countries show that the use of QIs correlates with improved five-year survival rates, earlier diagnosis, better adherence to evidence-based treatment protocols, and increased patient satisfaction. For example, in the Netherlands and Germany, the introduction of quality cancer care programs has led to improved surgical outcomes and overall survival for patients with colorectal cancer. The United Kingdom and Denmark have reported improvements in waiting times for diagnosis and treatment, and in Israel, screening rates for breast and colorectal cancer increased after the introduction of QIs for monitoring these conditions. The current review highlights the fact that countries with robust reporting systems and national cancer registries with high levels of data completeness, such as Denmark, Sweden, and Norway, were able to effectively monitor outcomes and adjust clinical practices accordingly. The findings suggest that implementing QIs in cancer care not only improves clinical outcomes but also promotes accountability and stimulates improved healthcare, ensuring better long-term patient care. This study highlights the value of adopting QIs as a global standard for assessing cancer care.

## Simple Summary

This review examines the impact of implementing quality indicators (QIs) in oncology care on patient outcomes. Quality indicators are a tool that, depending on the assumptions and strategies adopted, help to monitor a range of factors, including adherence to clinical practice guidelines, timeliness in the diagnostic and therapeutic process, safety of the procedures used, and changes in survival and mortality. The results of the inquiry suggest that despite the existence of significant differences in the healthcare systems analyzed, countries with strong reporting systems have achieved improvements in many areas. These include significant gains in five-year survival rates, earlier diagnoses, and improved treatment protocols. Overall, it may be concluded that the implementation of QIs leads to more effective oncology care, improving both clinical outcomes and patient satisfaction.

## 1. Introduction

**Figure 1:**
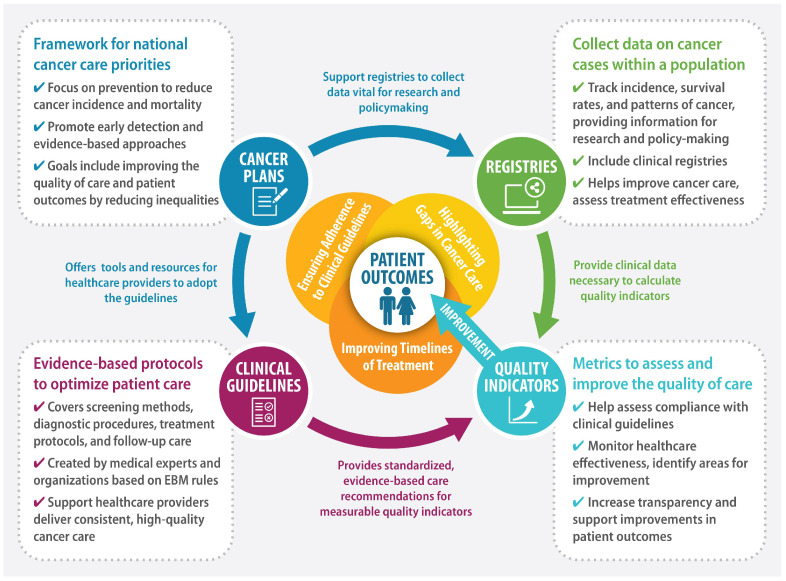
A framework for improving patient outcomes.

The World Health Organization (WHO) defines National Cancer Control Plans (NCCPs) as “public health programs designed to reduce the incidence and mortality of cancer and improve the quality of life of cancer patients through the systematic and equitable implementation of evidence-based strategies for prevention, early detection, diagnosis, treatment, and palliative care, making the best use of available resources” [1,2]. Effectively designed and implemented NCCPs can translate into improved cancer outcomes at the population level [3]. Figure 1 presents a framework for improving patient outcomes using QIs.

An important element of a high-quality healthcare system is that it should be based on valid and reliable data. Activities have been undertaken at national, international, and global levels to identify tools to improve the quality of healthcare services provided and empower patients in having their voice heard. These activities include the work of the National Quality Forum (NQF) in the USA, the Health Data Collaborative, and initiatives taken by the Organization for Economic Co-operation and Development (OECD), the Inter-American Development Bank (IDB), and the China Joint Study Partnership. Efforts to improve quality demonstrate that the process of assessing the quality of healthcare is of interest to both societies and governments around the world. High-income countries are investing in institutions in particular to strengthen the performance of health systems through regular measurement [4]. It is widely believed that quality of care (the efficiency and effectiveness of care) helps to minimize the exacerbation of disease, with the direct result of improved survival and quality of life for patients [5]. The tools used in the process of assessing the quality of healthcare may include, among others, oncology care. They are used in many different healthcare services to measure compliance with evidence-based standards established by national and international organizations. These indicators can be used at regional, national, and international levels [6]. However, the use of indicators at the international level requires the use of common methods for collecting, analyzing, and interpreting the obtained results. Most QIs in healthcare are theoretically derived from the quality framework defined by Donabedian, according to which they are divided into three categories: structure, process, and outcome [6,7,8,9]. Structural indicators refer to the environment (surroundings) in which care is provided: resources such as buildings, equipment, and staff with particular emphasis on their level of employment and qualifications. Process indicators refer to the provision of care, i.e., compliance with clinical guidelines, multidisciplinary coordination of care, or timeliness of the diagnostic and therapeutic process. Outcome indicators refer to the results of care received by patients and the general population: mortality, safety and adverse events, and patient-reported outcome measures (PROMs) [10,11].

There remain a variety of definitions of QIs in the scientific literature. QIs are most often defined as quantitative measures providing information on the effectiveness, safety, and/or patient-centeredness of care. Some institutions, such as the NQF in the USA, use the term “quality measure” instead of “quality indicator”. Others, such as the National Health Services (NHS) Indicator Methodology and Assurance Service and the German Institute for Quality Assurance and Transparency in Health Care (Das Institut für Qualitätssicherung und Transparenz im Gesundheitswesen), define additional characteristics with regard to indicators of quality. According to these definitions, indicators should provide information in relation to the following:

(a) A quality objective, i.e., a clear definition of the value (maximum or minimum) of the intended goal, depending on the indicator—for example, the mortality rate for patients admitted to the hospital with pneumonia should be as low as possible;
(b) A measurement concept, i.e., according to which the method for collecting data and calculating the indicator is defined—for example, in specifying the percentage of hospitalized patients diagnosed with pneumonia who died during their hospital stay;
(c) An evaluation concept, i.e., specifying how the measure is to be used to assess quality—for example, if the mortality rate of hospital patients is below 10%, this may be considered an indicator of high-quality care.

In the scientific literature, the terms “measures” and “indicators” are often used interchangeably. However, from the point of view of assessing the quality of care, it seems reasonable to use the term “quality indicator” for measures directly related to the concept of assessment. This is because measures that do not include the concept of assessment cannot attest as to whether the measured values represent good or poor quality of care. For example, the readmission rate is a measure of the number of times a patient is readmitted to the hospital, but it only becomes a QI when a threshold indicating a “higher than normal” rate of readmissions is established, which in turn may indicate poor quality of care [12,13]. QIs are an important tool for assessing the effectiveness and efficiency of both individual healthcare facilities and health systems as a whole. Where they have been implemented, differences in efficiency, safety, and patient-centeredness have been identified [14,15,16]. The use of QIs in the measurement of cancer care has allowed high-income countries (HICs) to develop initiatives for improving clinical practice (mainly at the hospital level) by helping to determine those areas in which the delivery of care is in need of revision, setting standards for best practices and providing the foundation for the introduction and development of new care processes. Additionally, publishing information about the indicator values achieved by individual facilities stimulates competition between care providers, which can also translate into improved quality of services provided [17,18,19,20,21,22].

Given that cancer is a leading cause of death and a significant obstacle to increasing life expectancy in all countries of the world, the development of tools for quality improvement is of fundamental importance. According to estimates by the WHO in 2019, cancer is the first or second leading cause of death before the age of 70 in 112 of 183 countries and is the third or fourth in another 23 countries. The burden of cancer morbidity and mortality is rising rapidly worldwide. This reflects both the aging and the growth of the population and changes in the prevalence and distribution of the main risk factors for cancer, many of which are related to socioeconomic development [23,24]. It is estimated that around 28.4 million new cases of cancer (including nonmelanoma skin cancer, excluding basal cell carcinoma) will occur worldwide in 2040, which is a 47% increase compared to the corresponding 19.3 million cases in 2020, assuming that national rates estimated in 2020 remain constant [23]. This translates into the need for continuous monitoring of quality through appropriate, territory-specific indicators [24,25]. Given the above, and the fact that countries have limited financial resources for healthcare, it is crucial to ensure that high-quality care in accordance with current standards is available to the widest possible population, regardless of age, sex, race, ethnicity, geographical location, religion, social status, language affiliation, or political beliefs [26]. However, in order to provide healthcare at the highest level, it is necessary to use appropriate tools in the process of assessing the quality of healthcare, and one such tool may be the QIs described here.

This cross-sectional literature review attempts to fill the gap in knowledge about the degree of improvement in the quality of oncological care in countries that have been systematically introducing and developing comprehensive solutions in health policy for many years, and that also promote systematic quality assessment using oncological care QIs. Its purpose is to analyze the programs, plans, and strategies for combating cancer, together with the implemented solutions that promote the use of QIs in the oncological care system, and to present the results of the activities conducted to improve the quality of care for patients with cancer. It may thus provide a useful introduction to further discussion on the development of good practices in the implementation of quality solutions in oncology. The aim of a comprehensive and focused review of this kind is to contribute to building more optimal solutions in the implementation of both QIs and cancer strategies. Thus, in addition to identifying areas in which further clarification of the effectiveness of existing solutions is needed, the present review may also guide future research with regard to the use of artificial intelligence (AI) algorithms in evaluating the quality of the care assessment process. Quality control programs are increasingly based on the use of AI, and inevitably it will be introduced on a large scale in healthcare systems over time. These algorithms can analyze huge sets of medical data to identify patterns or deviations, or even predict treatment outcomes. Currently, there is still a high degree of dispersion in the available data, along with inconsistencies in data collection methods, and the quality of the information obtained is often poor, giving rise to major limitations in the quality assessment process. Nevertheless, it is expected that in the coming years, in the context of monitoring the quality of oncology care, AI will be widely used to develop more precise and objective tools to assess the effectiveness of healthcare systems.

## 2. Materials and Methods

### 2.1. The Purpose of the Study

The aim of this review was to identify and synthesize findings from the available scientific literature, government reports, publications of scientific societies, and cancer organizations and associations with regard to the impact of assessing the quality of cancer care using QIs on improving cancer patient outcomes. Particular emphasis was placed on identifying countries that have implemented a systematic approach to assessing the quality of cancer care and on analyzing available data on the impact of these activities on treatment outcomes.

### 2.2. Search Strategy

The search for publications/documents/information included in this review was conducted in three stages.

#### 2.2.1. Stage I

In this stage, a literature search was conducted in the following databases: Medline, Embase, the Cochrane Library, and Google Scholar search engine, using the keywords “quality indicators” and “quality assessment” in order to identify countries that have experience with assessing the quality of oncology care using indicators. After a preliminary analysis of the sources identified, countries considered to have had a pioneering or leading role in the field of quality assessment of healthcare and, in particular, oncology care were selected for inclusion in the review. These countries are characterized by well-developed healthcare systems, the availability of high-quality data, and the development of national cancer control strategies or programs. In addition, these countries maintain active research on the quality of oncology care and have implemented various quality assessment systems. These selection criteria were designed to facilitate the process of comparing established quality assessment systems in countries that have prioritized cancer care in order to analyze experiences in assessing the quality of oncology care in different cultural and systemic contexts.

#### 2.2.2. Stage II

This stage involved a search of the government websites of the selected countries, focusing on institutions responsible for monitoring quality in healthcare and international organizations, including the World Health Organization (WHO), the Organization for Economic Co-operation and Development (OECD), the International Agency for Research on Cancer (IARC), and national cancer societies. The following keywords and their combinations were used at this stage: “national cancer plan”, “cancer strategy”, and “progress or performance reports”. In order to maintain consistency in the presentation of the results, the date of introduction of the first cancer plan in each of the countries under investigation was considered as the starting point for the examination of policy documents relating to the monitoring of quality in cancer care. For most countries, the starting date is in the early 2000s, but this is not the rule.

#### 2.2.3. Stage III

In this stage, the focus was on searching the pages of national cancer registries. Information was recorded on the origins and processes leading to the establishment of the registries, the scope of the data collected, and their usefulness in assessing the quality of oncology care.

In addition, a supplementary search of references meeting the review criteria was also conducted among the studies and reports included in the analysis.

### 2.3. Source Inclusion and Exclusion Criteria

#### 2.3.1. Included in the Review:

Types of sources: scientific articles, government reports, reports of scientific societies, organizations, and associations fighting cancer.

Topic: sources regarding the impact of assessing the quality of oncological care using QIs on the treatment outcomes of oncological patients.

Publication period: all available publications up to 20 February 2025.

Language: there were no restrictions on the language of the studies retrieved, in view of the fact that national programs are usually published in the official language of a given country.

Countries: the USA, Canada, the UK (England and Scotland are described separately because of the differences between the Scottish and English healthcare systems), the Netherlands, Germany, France, Italy, Spain, Sweden, Denmark, Norway, Israel, Japan, and Australia.

#### 2.3.2. Excluded Sources:

Those not specifically related to the subject of the study. These included abstracts, letters, comments, and opinions.

## 3. Results

At the outset, it should be noted that direct identification of the impact of implementing individual solutions and linking specific pro-quality tools with the achieved results in an unambiguous way is almost impossible due to the variety of factors that may affect the results and the constant development of both medicine and systemic solutions. In addition, the study did not focus exclusively on the latest initiatives in order to capture both changes in health priorities over the years and the impact of the programs on the quality of services provided. This approach allowed for a more in-depth understanding of the long-term impact of individual programs on the quality of care and monitoring their effects in a changing technological and systemic context. It also allowed for the identification of trends and a better means of linking any improvements in the quality of healthcare with the actions to combat cancer implemented in the individual countries included in the review. This influenced the presentation and descriptive nature of the presented results.

### 3.1. USA

Since the signing of the National Cancer Act in 1971 which established the National Cancer Program, the United States has initiated broad-based efforts to combat cancer. The National Cancer Act, known as the “war on cancer”, entrusted the director of the National Cancer Institute (NCI) to coordinate governmental and non-governmental efforts in combating the disease. Significant advances in cancer care have resulted in the development of tools for earlier diagnosis, less toxic therapies, improved quality of life for survivors, and more equitable distribution of the benefits of research [27,28]. Over the past 30 years, numerous programs have been implemented to improve the quality of care, increase access to treatment, and reduce cancer-related mortality [27,28]. One notable example is the National Comprehensive Cancer Control Program (NCCCP), launched by the Centers for Disease Control and Prevention in 1998. The purpose of this program was to integrate efforts aimed at cancer prevention and control. Its mission was to foster collaboration among various sectors, including government agencies, community organizations, and healthcare institutions. Another significant step was the Quality Oncology Practice Initiative (QOPI), launched by the American Society of Clinical Oncology (ASCO) in 2002. The QOPI aimed to improve the standards of oncology care by establishing quality measures that would allow for the assessment and self-improvement of medical practices. The QOPI provided physicians with data that allowed for comparison of their results with other facilities, creating a framework for identifying areas requiring improvement [29,30]. Another example of pro-quality activities are QIs developed by the Agency for Healthcare Research and Quality (AHRQ). Currently, these indicators function in four areas, i.e., preventive quality indicators (PQIs), inpatient quality indicators (IQIs), patient safety indicators (PSIs), and pediatric quality indicators (PDIs). PQIs are intended to identify conditions that can be controlled by effective outpatient care. IQIs assess the quality of care within hospitals and identify areas requiring improvement. The role of PSIs is to assist hospitals in assessing the incidence of adverse events and complications in hospital care and identify areas that may require corrective action. PDIs, on the other hand, focus on detecting deficiencies in pediatric care [31,32]. These AHRQ quality indicators (AHRQ QIs), which are standardized, evidence-based measures of healthcare quality, provide a means for measuring and tracking clinical outcomes. They are an essential tool in the armamentarium of decision-makers for evaluating data, drawing attention to potential problems with quality issues, identifying those areas in need of further evaluation and analysis, and recording changes over time. They are reported at both the hospital and the district level and are available to users through free software. At the hospital level, they are used both to support quality improvements within facilities and to track and evaluate adverse events related to patient safety. At the district level, they are used to identify access to outpatient care that includes appropriate follow-up care after hospital discharge. These tools are thus intended to support the development of trend and standards information in order to compare a hospital’s current QI performance with previous years (trend) and with national benchmarks [32].

In 2016, the so-called “Cancer Moonshot” was launched, which according to the creators of the program, was an initiative aimed, among other things, at contributing to accelerating scientific discoveries in cancer research and supporting activities in the field of collecting and sharing data on cancer in the US. The program created conditions for cooperation between various interest groups, including patients, researchers, and clinicians, in an effort to improve the situation of cancer patients. Currently, those involved in a subsequent phase of the project which began in 2022, have adopted the goal of reducing the rate of cancer deaths by half over the next 25 years [33].

**Figure 2:**
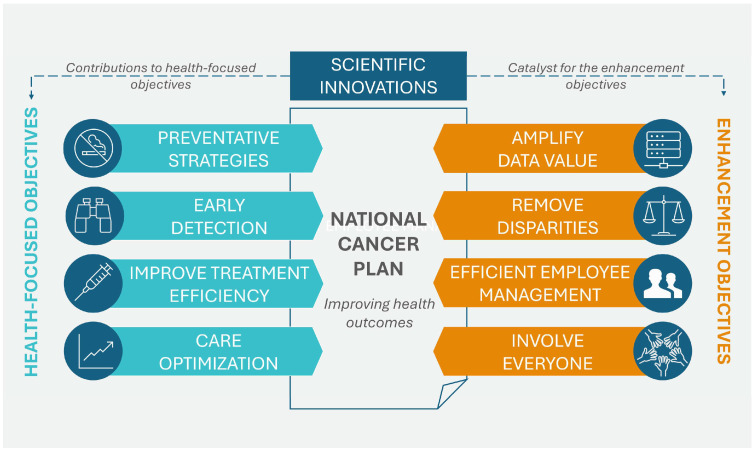
Goals of the U.S. National Cancer Plan. Source: [28].

In April 2023, the NCI of the U.S. Department of Health and Human Services published the National Cancer Plan. The plan, consisting of eight main goals, aims to unite the oncology community in a coordinated effort to prevent cancer and improve diagnosis, treatment, and quality of life for patients. These goals can be divided into two groups: the first group addresses health-centric goals, which include prevention and early detection, development of effective therapies, and ensuring optimal levels of care, while the second group concerns empowerment goals, which include increasing the usability of data, eliminating inequalities, optimizing human resources, and engaging people as widely as possible in the overall endeavor of overcoming cancer. Additionally, the program promotes support for innovation and developing new technologies that will be the driving force for the formulated goals. Figure 2 shows the eight goals that form the basis of the U.S. cancer plan [28].

According to information included in the latest Cancer Plan, the planned programs have contributed to reducing cancer-related mortality by one third (a decrease of approximately 33%) over the last three decades. It is worth noting that within the framework of the Cancer Moonshot program it was optimistically assumed that this trend would not only continue, but would accelerate, reducing cancer mortality by 50% by 2047 [28]. Programs such as the NCCCP and the implementation of systematic quality measures, including QIs developed by the AHRQ, have contributed to better coordination and improvement of healthcare. On the basis of this experience, it can be concluded that a comprehensive approach to cancer control and prevention, based on public and private sector cooperation, the introduction of innovations, and the implementation of QIs, has been the foundation of strategies to further reduce the societal burden of cancer and improve the quality of life of patients [27,28].

An action worthy of note in the drive to improve survival rates is the creation of cancer registries. In the USA, an initiative of this kind was launched in 1971 in the form of a system of cancer registries operating in selected states (the data gathered covered approximately 30% of the population and allowed around 400,000 new cases of cancer to be registered per year). The main goal of the registries was to collect data on patient demographics, characteristics of cancers, diagnostics, and implemented treatment. As a result of these activities, the National Program of Cancer Registries (NPCR) was established, which had the clear benefit of providing appropriate levels of funding and specialized assistance to improve existing technical solutions and registries. Standards for the completeness, timeliness, and quality of data were put in place, and assistance and training was provided in the establishment of a computerized system for reporting and processing data. As indicated in a review by White et al. [34], the NPCR covered approximately 96% of the U.S. population and recorded more than 1.6 million newly diagnosed cancer cases per year. According to the authors of the review, data from cancer registries provide a basis for public health initiatives aimed at reducing disparities in cancer incidence, mortality, and survival. Furthermore, the data can be used to define and monitor cancer incidence at the local, state, and national levels, as well as examine cancer treatment patterns or assess the effectiveness of public health efforts aimed at prevention.

One example of using indicators to assess adherence to guidelines is the study by Shapiro, Zubizarreta, and Moshier et al. [35] on the quality of care following cancer survival. The authors investigated the implications of the recommendations from the QOPI guidelines for the period after cancer survival for routine clinical practice. Six measures were selected for the study that were related to cancer survivorship and included 1. a recommendation to stop smoking; 2. provision of advice or referral for stopping smoking; 3. a discussion of the risks of infertility after chemotherapy; 4. referral to fertility specialists; 5. performance of a PET, CT, or bone scan within 12 months of breast cancer diagnosis for patients treated with the intent of achieving total remission; and 6. monitoring of tumor markers within 12 months of breast cancer diagnosis in patients treated with curative intent. The selected measures were assessed between 2015 and 2019. The authors found that the recommendation to cease smoking and the provision of advice or referral for smoking cessation increased over time from 50% to 61% and from 34% to 50%, respectively. Discussion of infertility risk before chemotherapy increased from 36% to 53% and discussion of fertility issues or referral to fertility specialists increased from 23% to 38%. In 29% of women diagnosed with early breast cancer who received treatment with the aim of total remission, a PET scan, CT scan, or bone scan was documented within the first year following treatment completion, and this measure did not change over the study period. Tumor marker monitoring, however, increased from 78% to 88%. The authors emphasized the importance of the guidelines and, in particular, their translation into clinical practice, along with the use of markers for monitoring results, and they were confident that these would increase as evidence for the effectiveness accumulates.

### 3.2. Canada

Modern, integrated cancer control efforts in Canada began to be planned in the late 1990s. Since 1999, Health Canada, in collaboration with the Canadian Cancer Society, the National Cancer Institute of Canada, and the Canadian Association of Provincial Cancer Agencies, has been working work on the Canadian Strategy for Cancer Control (CSCC). The strategy has been introduced systematically since 2006 to optimize the benefits of existing knowledge and resources in cancer control, while increasing the sustainability of the healthcare system through more integrated planning, prioritization, development, and implementation of public policy. The primary goals of the CSCC were to improve prevention measures, thereby reducing the number of people diagnosed with cancer, providing earlier diagnoses, improving the quality of life of people living with cancer, and reducing the death rates associated with the disease. The CSCC introduced a more integrated and comprehensive approach to healthcare management. It built on the strengths of Canada’s federal and provincial health systems, creating pan-Canadian networks of experts to drive action and leverage knowledge and experience. A knowledge transfer and risk management platform has been established to provide governments, non-governmental organizations, and individual Canadians with the information and tools they need to make informed decisions about cancer risk and control. The strategy was designed to be flexible and accountable to ensure that cancer control priorities were met as they emerged. The initial priority areas for 2006–2010 include prevention and early detection of cancer, patient support on the diagnostic and therapeutic path, support for medical personnel, development of cancer research, and improvement of the provision and availability of information on cancer [36].

As stated in the 2004 Progress Report on Cancer Control in Canada, the expected outcome was to be the integration and coordination of activities within the CSCC, with a view to reducing cancer incidence, morbidity, and mortality and improving the quality of life of people with cancer. As this report indicates, Canada’s response to this challenge has been generally positive, and significant progress has been made over the past 15 years. Small declines in cancer mortality rates for both men and women have been observed since the late 1980s, primarily due to declines in mortality rates for breast, prostate, and colon cancers. Perhaps the most important progress in this area has been the decline in cigarette smoking, which has led to declines in lung cancer rates in men. As the report suggests, progress in these areas can be attributed to broad societal trends, as well as specific initiatives in cancer prevention and advances in cancer screening and treatment [37].

Another document, the 2011 Cancer System Performance Report, presented indicators used to measure health system performance and identified areas for improvement. The key findings of the report are presented below, divided into seven categories:

1. Prevention—There was a decline in smoking and exposure to passive smoking. At the same time, there was an increase in alcohol consumption and in the percentage of Canadians who were overweight or obese. Attention was also drawn to the differences in the uptake of HPV vaccinations across provinces.
2. Screening—There were no significant differences in Pap test participation rates, but significant variation in colon cancer screening participation rates by province (ranging from 22% to 52%) was noted, probably due to differences in the start dates of each provincial program.
3. Diagnostics—Provincial cancer registries noted an increase in the percentage of cancer cases reported, with stage data (six out of nine provinces reported an increase of 90% or higher). However, the time from a positive mammogram to diagnosis (for breast cancer) continued to vary significantly across the provinces (the percentage of cases diagnosed within the target time frame ranged from 38% to 84%).
4. Treatment—The target for starting radiotherapy within four weeks of being qualified for treatment in 90% of patients was reached in seven of the ten provinces. There was also an increase in radiotherapy efficiency in 2010 over 2009, as measured by the number of accelerators per capita. Additionally, the rate of radiotherapy use, while relatively consistent across provinces (29% to 34%), continued to show a downward trend with respect to patient age.
5. Research—Adult participation rates in clinical trials across the provinces ranged from 1% to 8%, with declining rates for children reported in seven of the eight provinces that submitted data.
6. Patient experience—In terms of patient satisfaction with coordination and continuity of care, there was a considerable range in positive experiences reported of 50% to 90%. The area of “provider awareness of medical history” was reported as being least satisfactory across all the provinces, while “knowing who is responsible for each treatment” achieved the highest evaluation.
7. Long-term results—It was found that from 1995 to 2007, the overall incidence rates for cancer in men remained stable, but in women they showed an increase. Moreover, the overall cancer mortality rates had decreased markedly in men, but less so in women. The authors of the report indicated that these patterns were largely attributable to lung cancer, where incidence and mortality rates decreased by 20% in men but increased by 8% in women between 1992 and 2007 [38].

**Figure 3:**
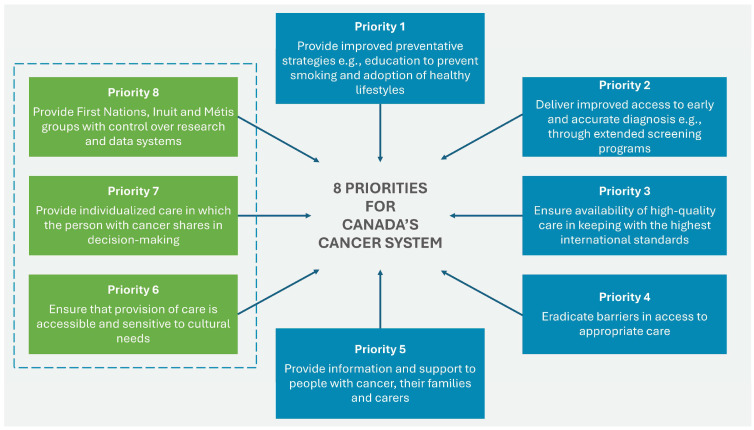
Eight priorities for Canada’s cancer strategy 2019–2029. Source: Canadian Strategy For Cancer Control [39].

The 2019 Canadian Strategy for Cancer Control is a refreshed and updated version of the 2006 Strategy and presents a 10-year action plan to improve inequities in the cancer care system. The updated document identifies eight priorities (see Figure 3) with specific actions to strengthen cancer care for all Canadians, families, and caregivers of those affected by the disease.

Priorities 6 to 8 were identified by First Nations, Inuit, and Métis organizations and communities. For each of these priorities, actions specific to First Nations have been identified to help drive the necessary changes in outcomes and experiences of all ethnic groups [40]. The four main goals of the strategy are focused on:

- Equal access to high-quality care for all residents;
- Reducing cancer rates;
- Increasing the number of people who survive cancer;
- Improving the quality of life of people suffering from cancer [39].

In the introduction to the Canadian Strategy for Cancer Control 2019–2029, emphasis is placed on the fact that efforts to reduce the burden of cancer since the implementation of the strategy have resulted in significant progress in prevention, diagnosis, treatment, and palliative care. From 2007 to 2018, survival rates improved for most cancers, including breast, colon, and lung cancer, with more than 65% of cancer patients being alive five years post diagnosis. There were changes in the mortality rate from prostate cancer, which fell by 23%, and in breast cancer by 22%. There was also a 15% decrease in the number of cases of lung cancer and a 17% decrease in the number of cases of colorectal cancer, which is attributed to effective prevention and screening [40]. One of the studies indicating Canada’s progress in the fight against cancer is the analysis by Warkentin et al. (2023) focusing on an attempt to estimate the number of deaths avoided, in an analysis of data since 1988. According to the published results, the authors of the study indicate that in Canada since 1988, approximately 492,629 deaths due to cancer have been avoided (372,584 among men and 120,045 among women), which indicates a reduction in overall mortality of 24% for men and of 10% for women in the period studied, which, as they emphasize, is significant despite the observed increase in the total number of cases resulting from the growth and aging of the population [41].

Although the first cancer registries in Canada date back to 1932, the most important is the Canadian Cancer Registry (CCR), established on the basis of the National Registry Database in 1992 and managed by Statistics Canada. It fulfils an essential role in the healthcare system, as all newly diagnosed cancer cases in Canada are registered by the CCR, and by virtue of collecting information on diagnosis, stage of cancer, treatment, and the results of therapies implemented, the CCR is the most important source of data used for epidemiological surveillance, cancer research, healthcare system planning, and evaluation of prevention programs, providing support for the healthcare system. Additionally, the CCR, in cooperation with provincial registries, sets national standards for data collection and quality management [42,43].

### 3.3. United Kingdom (England and Wales)

As a result of many years of efforts to improve the delivery of healthcare by the NHS, and in response to issues such as high mortality and inequalities in access to treatment, the first national cancer program, the NHS Cancer Plan, was finalized in 2000. This document was created as part of wider reforms to the NHS, initiated by the NHS Plan, which focused on investment in and modernization of the healthcare system in the UK. The plan consisted of a number of key assumptions, including investment in staff, equipment, and technology, as well as the development of screening and diagnostic programs; promoting a healthy lifestyle, with an emphasis on reducing smoking and promoting a diet rich in fruit and vegetables; reducing waiting times for diagnosis and treatment; and eliminating inequalities related to access to health services. An additional objective as part of the plan was to implement standards of care and to monitor progress in improving quality through dedicated audits. The importance of appropriate communication with patients to enable their involvement in the decision-making process was also emphasized [44]. Three new commitments were made as part of the plan. The first of these concerned intensifying actions to further reduce smoking among adults; the plan determined to achieve a reduction in the percentage of smokers from 32% in 1998 to 26% in 2010. The second commitment concerned shortening the waiting time for diagnosis and treatment so that patients would not wait longer than a month from the moment of referral to the start of treatment. The third commitment included additional investments in palliative and hospice care [44].

Almost at the same time as the first cancer plan was published, the National Institute for Clinical Excellence (NICE) was established in April 1999. Its aims included creating consistent guidelines that could be used across the country. On 1 April 2005, the Institute merged with the Health Development Agency to become the new National Institute for Health and Clinical Excellence (the acronym NICE remained unchanged). From 2009, NICE was responsible for developing and reviewing indicators for the Quality Outcomes Framework (QOF), and after April 2013, NICE changed its status from a special health authority to a non-departmental public body and became the National Institute for Health and Care Excellence (the acronym NICE continued to remain unchanged). It also formally undertook work to introduce evidence-based guidelines and standards into the social care sector, in accordance with the Health and Social Care Act 2012. In 2018, NICE published a report on the impact of cancer on patients and the healthcare system. The report (NICE impact—cancer) considered how evidence-based guidelines can help improve the diagnosis and treatment of cancer. This was the first of several impact reports that looked at how the health and social care system was using NICE recommendations to improve outcomes in priority areas [45].

The plan contributed to maintaining the downward trend in cancer mortality rates in England. Mortality rates were found to be falling faster than the predicted trajectory set by the Department of Health, meaning that the plan’s target of reducing mortality by 20% among those under 75 by 2010 was close to being achieved. As the report “The NHS Cancer Plan: a progress report” showed, despite a 31% increase in cancer incidence between 1971 and 2000 in terms of new cases per 100,000 population, by 2002 the cancer mortality rate had fallen by 12% (18% for men and 7% for women). The report cited the introduction of national screening programs and new treatments as the direct cause of the fall in mortality rates. Furthermore, access to cancer diagnosis and treatment was greatly improved, and progress had been made towards meeting the targets for maximum waiting times for diagnosis and treatment. As indicated in the report, more than 99% of patients referred urgently by a GP were seen by an oncology specialist within two weeks. Other successes included the implementation of multidisciplinary teams (MDTs) and the introduction of standards of care [46,47].

In discussing quality issues, it is important to mention the establishment of the National Cancer Registration and Analysis Service (NCRAS) in 2013, which was designed to be a single unit for analyzing cancer data across England. It was created through the combination of eight regional cancer registries and data systems that had been covering cases since 1971. The NCRAS, which operates as a population-based cancer registry, was initially part of Public Health England (PHE), but in 2018 it was integrated into the National Disease Registration Service (NDRS), and in 2021 its management was transferred to NHS Digital. In 2023, in order to meet the demands of a better-integrated care system, control of the data held by the NDRS was transferred to NHS England (NHSE). The main objective of these initiatives has always been to further improve the collection of high-quality data. The NCRAS provides detailed, comprehensive data on cancers diagnosed in England, enabling monitoring of the entire cancer care pathway, from diagnosis to treatment and health outcomes. These data are collected from a variety of sources, including multidisciplinary team meetings, pathology reports, molecular test results, and hospital administration systems. Data collected by the NCRAS are used nationally and internationally for epidemiological studies, healthcare planning, clinical audit, and comparison of outcomes. In addition, the NCRAS collaborates with a variety of healthcare providers in England to support ongoing monitoring and assessment of the quality of care. Advanced validation and data quality processes ensure that the registry provides reliable and complete information on cancer cases, supporting data-driven decision-making. High data quality in the registry is achieved through manual and automated checks, which contribute to the accuracy and timeliness of cancer registrations. A significant success of the NCRAS is the reduction in errors, with fewer than 0.1% of errors detected over 10 years, which, as indicated by Henson et al. [48], indicates very high data quality. Additionally, NCRAS data for 2019 were the source of over 260 scientific publications and the development of statistics on morbidity, survival, and mortality, which can contribute to making appropriate decisions in health policy.

As part of the NHS Long Term Plan, which was published in 2019, in addition to the two main goals for cancer, three main priorities were set out to help achieve these goals (see Table 1).

The “NHS England Cancer Programme Progress Update—Spring 2024” (published in May 2024) reported on progress made under the current program, summarizing the work undertaken under the 2019 plan. The plan had two key targets, namely to increase the proportion of cancers diagnosed at stages 1 and 2 from around half to 75% by 2028, and to increase the number of people surviving for at least five years after a cancer diagnosis. The authors of the report emphasized that, to the best of their knowledge, no country in the world had achieved such a high proportion of stage 1 and 2 diagnoses. The report showed that by March 2024, the NHS had met the targets set out in the plan. At least 75% of patients had received a definitive diagnosis or a diagnosis of cancer was ruled out within 28 days. The number of people with suspected cancer waiting more than 62 days for services after an urgent referral had also been reduced. The report also described the first significant increase in early diagnosis rates in a decade [49].

### 3.4. United Kingdom (Scotland)

The first national strategy to improve cancer care in Scotland was “Cancer in Scotland: Action for Change”, published on 3 July 2001. It set out a direction for the development and improvement of cancer services in Scotland over the coming years. The strategy defined a wide range of actions that were needed to continue to find ways to prevent cancer, allow for its early detection, and improve the treatment and care of people with cancer in Scotland. As part of this initiative, efforts were also directed towards the establishment of regional cancer advisory groups (RCAGs) and managed clinical networks to improve the coordination and quality of cancer care by ensuring consistent standards and better communication between regions. It is important to note that the regional organizational structures were intended, among other things, to drive and implement the cancer strategy. As described in “Cancer in Scotland”, three RCAGs were established for North, West, and Southeast Scotland, each providing a strategic, advisory, and planning focus for the relevant local cancer services and NHS boards [50].

In February 2004, the fourth monitoring reports of “Cancer in Scotland: Action for Change” were published. These reports summarized the period from April 2003 to September 2003 for the three areas of Scotland under scrutiny, and included progress on investments implemented in pursuit of the realization of the cancer plan from 2001 to 2002 (updating the first monitoring reports) and progress on investment/implementation plans from 2002 to 2003 (updating previous monitoring reports). The reports identified a number of areas in which success had been achieved, including:

- Speeding up access to diagnosis and treatment: a rectal bleeding clinic in the Highlands maintained the maximum waiting time for an outpatient appointment at 2–3 weeks; a clinic in Lanarkshire, where the percentage of patients receiving an appointment within two weeks and notification of results within a further two weeks, achieved a standard of 80% among patients);
- Improving treatment and care for patients: clinics run by nurses specializing in breast cancer freed up 30–40 places in other clinics, helping to provide an improved service to patients; increasing the volume of brachytherapy services, thereby increasing patient satisfaction;
- In palliative care: an increase in palliative care services in Tayside nursing homes resulted in a reduction in the number of admissions to acute services);
- Investment in staff and technology: in Lothian, the appointment of an additional breast cancer surgeon enabled the national target for waiting times for breast cancer treatment to be achieved, with 87% of patients starting treatment within 1 month of diagnosis; in Dumfries and Galloway, the provision of equipment to automate the analysis of laboratory results improved access to results, reducing the time needed to complete charts manually from 5 h to 1 h [51].

Subsequently, in 2004, the Cancer in Scotland: Sustaining Change strategy continued the original approach, aiming to maintain achievements and ensure equal access to treatment across Scotland. The introduction of quality standards for different types of cancer was intended to help reduce waiting times for treatment, providing a more standardized approach to care. In 2008, a further strategy, “Better Cancer Care: An Action Plan”, was implemented, which was focused on prevention, early detection, and improving the quality of life of patients. It continued the work on the approach to smoking that began in 2006 with a ban on smoking in public places, and in 2007, legislation was introduced to raise the age of sale of tobacco from 16 to 18. “Scotland’s future is smoke-free: A Smoking Prevention Action Plan”, published in May 2008, set out a program of action to discourage children and young people from taking up smoking and becoming regular smokers by making cigarettes and other tobacco products less attractive, affordable, and accessible. The plan also included an expansion of breast, bowel, and cervical cancer screening programs to detect cancers at an early stage. The main areas of the plan included prevention and early detection, development of genetic and molecular tests, improvement of the referral system and diagnostic process (including increased access to PET), improvement of the therapeutic process (with particular reference to surgical treatment, laparoscopic surgery in the case of colon cancer, and optimization in the field of chemotherapy, radiotherapy, and the development of new drugs), and support for patients during and after treatment (encouraging return to the labor market, psychological support, palliative care, etc.). All these activities were to be focused on a patient-centric approach and translate into an improvement in the quality of care for oncological patients [52]. As indicated in the report entitled “Better Cancer Care: An Action Plan”, the implementation of these strategies brought significant benefits. Investments in diagnostic technologies and improvements in the availability and quality of tests increased the opportunities for quick and effective diagnoses. Screening programs enabled cancers to be detected at earlier stages, which meant that effective treatment could be instituted sooner and resulted in higher survival rates. In addition, subsequent information and education campaigns, along with the legislative changes that accompanied them, such as the ban on smoking in public places, contributed to a reduction in the incidence of smoking-related diseases, including lung cancer. The new “straight-to-test” diagnostic system has enabled patients with suspected bowel cancer to proceed quickly from referral to testing, which has significantly shortened the time to diagnosis and treatment. For example, in the NHS Forth Valley region, the waiting time for a CT scan was shortened from 12 to 6 weeks, which significantly sped up diagnosis. Moreover, these changes influenced the overall improvement of the quality of cancer care in Scotland, and the results are reflected in higher survival rates, faster diagnosis, and wider access to modern treatment methods. Between 1980 and 1984 and between 2000 and 2004, there was an improvement in survival at five years after diagnosis, which was especially significant for some cancers. For example, in men, survival for malignant melanoma of the skin rose from 62% to 87% between the two periods, an absolute increase of 25%. This is most likely attributable to achieving earlier-stage diagnoses in the wake of health education programs aimed at encouraging earlier presentation and referral. Considerable absolute increases in survival rates at five years were also observed for men and women: for colorectal cancer 19% and 18%, for Hodgkin’s disease 20% and 27%, and for leukemia 27% and 25%, respectively.

According to the 2008 strategy for Scotland, the previous decade had seen an overall decline in mortality rates for cancer, which had fallen by 12% in men and 5% in women. In men, the largest declines were for stomach, lung, and colon cancers (29%, 22%, and 19%, respectively). A decrease of 9% in deaths from prostate cancer had also been observed, this being the second most common cancer diagnosed in men in 2005. In women, stomach and colon cancers (37% and 17%, respectively) showed the largest declines. Mortality rates from breast cancer, the most commonly diagnosed cancer in women, fell by 12%, despite an overall increase in breast cancer cases. Cervical cancer mortality also fell by 32%. It was reported that in 2005, colorectal cancer accounted for 14.5% of all cancers in men and 11.3% in women. Here too, five-year survival rates demonstrated significant improvements, increasing from 35% for patients diagnosed in 1980–1984 to 55% in 2000–2004. The document also set out a broad approach to improving the quality of cancer care in Scotland, in accordance with a number of key principles: care should be patient-centered, safe, effective, efficient, equitable, and timely. The “Better Together” patient feedback program in Scotland, also launched in 2008, prioritized an approach based on patient feedback. Its aim was to improve cancer services by actively “listening” to patients’ needs and responding to their reported experiences [52].

In April 2011, Healthcare Improvement Scotland (HIS) was established as part of the Scottish NHS. HIS is the national organization for improving healthcare in Scotland. Its primary aim was to enable people in Scotland to receive the best quality health and social care, and one of the organization’s key roles was to develop cancer quality performance indicators (QPIs). The QPIs were developed in partnership with clinicians from the three regional cancer networks and Public Health Scotland Data and Intelligence. The QPIs were to ensure that NHS boards focused on the areas that matter most to patients. This included improving patient survival rates, improving patients’ experiences of care, reducing variation in care between regions, and delivering patient-centered cancer care [53].

In March 2016, the Scottish Government published the next stage of its strategy: “Beating Cancer: Ambition and Action”. The strategy focused on improving cancer prevention, detection, treatment, and follow-up care in Scotland. The document placed particular emphasis on reducing healthcare inequalities and ensuring patient-centered care, with a focus on enabling patients to navigate the healthcare system efficiently. The challenge for the future was to develop sustainable, innovative models of care to meet complex needs, provide high-quality support, and improve survival outcomes, especially in communities with restricted access to healthcare. The strategy placed an emphasis on a patient-centered approach by encouraging active patient participation in decision-making [54].

The latest strategy, Cancer Strategy for Scotland 2023–2033, continues the Government’s work to improve cancer care in Scotland. It is based on 11 key ambitions/areas, including prevention, early detection, better preparation for treatment, ensuring safe and effective treatment, improving care before and after treatment, developing workforce capacity, delivering patient-centered care, tackling inequalities in access to services, providing psychological support as part of primary care, developing research and innovation, and developing IT services to support cancer communication (see Table 2). It also focuses on the four key principles of patient-centered care: compassion, personalization, coordination, and enabling, which underpin the approach set out in the document. The strategy aims to improve survival rates by 2033, particularly for cancers that currently have lower survival rates, such as lung cancer. A number of public health interventions have been planned, which, in the opinion of the document’s authors, should have greater preventative effects and increase access to high-quality services, which will consequently improve survival rates and reduce disparities in oncological care between regions [55].

It is planned that the Scottish Cancer Network (SCN) will act as the delivery arm of the strategy. In addition, the SCN will continue to play a leading role in defining clinical pathways from diagnosis. These pathways will cover treatment and care through to the end of life, defining clinically agreed-upon best practice and providing common standards of care for people with cancer, regardless of where they live. The SCN will also link up national cancer networks, where national integration and collaboration in specific cancers can best utilize expert resources and improve outcomes for people with cancer. It will also lead the work of the NHS Scotland “Once for Scotland” workforce policies program, which is designed to review and transform existing workforce policies and work closely with regional networks, where this work is better carried out at a more local level. This work will be supported by the Scottish Cancer Quality Program, which will set out quality requirements using agreed quality performance indicators (QPIs) that meet the objectives set out in the strategy. Key clinical indicators linked to the strategic objectives will be closely monitored in partnership with Healthcare Improvement Scotland [55]. A detailed monitoring and evaluation framework has been developed as part of the Strategy 2023–2033 and the Cancer Action Plan 2023–2026. An annual progress update will summarize key actions. As part of monitoring the implementation of the strategy, three evaluation reports will also be produced: the first three-year evaluation report will be published at the end of the first action plan in autumn 2026, the second at the end of the next three-year period of the plan in 2029, and the last, summarizing all actions taken under the strategy, will be produced at the end of the strategy period [56].

The first annual progress update report was published in July 2024. Progress was reported in ten of the eleven areas set out in the strategy and referred to as “ambitions” with equal priorities. On the understanding that addressing health inequalities is a key driver for all actions, Ambition 8 of the strategy (Tackling Inequalities) is included within the other ambitions and is therefore not included in a separate section. In view of the fact that the paper is concerned with QIs and that further development of them is planned under Ambition 11 (Cancer Information and Intelligence-Led Services), the focus of the report is on progress in this area only. It recommends the further development of quality performance indicators and identifies them as a key driver in the overall quality improvement program for cancer services. In addition, the progress report indicates that 30-day mortality data in systemic anti-cancer therapy (SACT) were first published in July 2023, and a new SACT activity dashboard was launched in October 2023 and has been published weekly since then. A basic framework for collecting data about PROMs was also developed. The report highlighted that the SCN continues to develop new diagnostic and treatment pathways, which are published on the “Right Decisions” website. A resource for the general public has also been launched [57].

Efforts to establish and maintain cancer registries are also worth mentioning. The main example is the Scottish Cancer Registry, which is an important complement to efforts aimed at improving all outcomes in the field of cancer care. The registry has been collecting data on cancers since the late 1950s and currently contains information on over 1.8 million cases. In accordance with the adopted methodology, various types of information are recorded for each case, such as patient demographic data and diagnostic data, including tumor location and histological type. Since 1997, the database has been converted from a tumor-based database into a patient-based database, which directly translated into the collection of additional, more specific data on diagnosed cancers, implemented treatment, and outcomes. According to information published on the website of the Scottish Public Health Observatory, a total of around 55,000 new diagnoses of all types of cancers are registered each year, including in situ conditions, benign tumors, and tumors of uncertain nature [58,59,60].

### 3.5. The Netherlands

**Figure 4:**
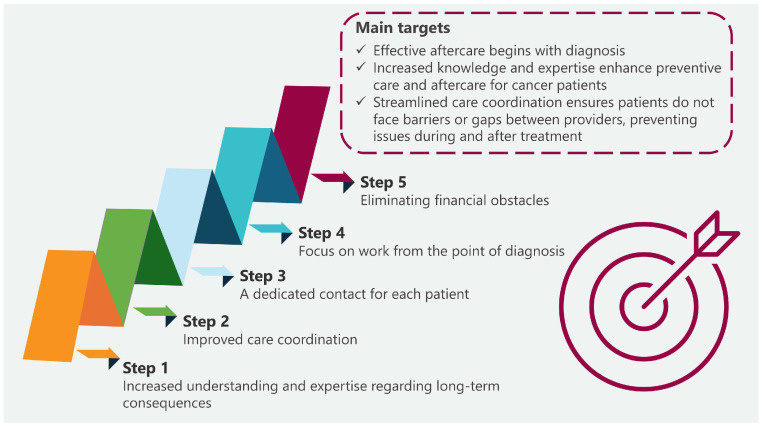
The main objectives of the Dutch National Action Plan Cancer & Life. Source: Integraal Kankercentrum Nederland https://iknl.nl/kanker-en-leven/nationaal-actieplan-kanker-leven (accessed on 29 October 2024).

The Netherlands National Cancer Control Program for the years 2005–2010 was introduced in November 2004 as a response to the increasing burden of cancer. The program was established by the Ministry of Health in cooperation with the Netherlands Federation of Cancer Patients’ Organizations, the Netherlands Cancer Society (Kankerbestrijding), and major oncology centers (Association of Comprehensive Cancer Centers). The aim of this program was to create a coherent and uniform system that would allow for more effective management of cancer prevention, diagnosis, and treatment. The program focused on seven priority areas: reducing tobacco smoking, implementing screening tests for colon cancer, improving the quality of oncological care, developing psychosocial care, and educating specialists in oncology. The aim was to improve the health of the population, reduce the number of cases, increase the chances of survival and improve the quality of life of oncological patients and their families. From the information included in the report entitled “Progress Report on Cancer Control in the Netherlands” published by the Netherlands Ministry of Health, it can be concluded that, despite not fully achieving the expected decrease in the number of smokers, significant progress was made as a result of the implementation of the plan. A psychosocial support system was also developed, creating guidelines and making use of instruments such as the “Distress Thermometer” to assess patient needs. A consumer quality (CQ) index was also used to assess patient satisfaction with oncological care, with the aim of systematically monitoring patient satisfaction and improving the quality of care provided in the future. Additionally, an increased 5-year survival rate was achieved, which improved from 47% (for patients diagnosed in 1989–1993) to 59% in 2004–2008, and which, according to the program assumptions, was an improvement of 12 percentage points. It also proved possible to maintain an 80% attendance rate for breast cancer screening tests, which represented a slight increase over the duration of the program. The authors of the report also indicated that the percentage of referrals for additional tests in the case of breast cancer increased (from 14 per 1000 women examined in 2004 to over 18 per 1000 in 2008), which might have contributed to better detection of cancer in the early stages and allowed for faster implementation of treatment. Furthermore, the report indicated that a larger number of facilities cooperating within the NCCP reported improvements in the implementation of standards, which had a significant impact on the quality of care (no direct numerical data were provided). At the same time, the NCCP also contributed to improved cooperation between institutions and organizations responsible for oncology activities, enabling more integrated and effective oncological care, which increased the availability and quality of care and reduced delays in diagnosis and treatment [61]. Figure 4 shows the main objectives of the National Action Plan Cancer & Life (Nationaal Actieplan Kanker & Leven).

Another initiative aimed at improving the quality of oncological care was the “Quality of Cancer Care” program, which was launched in 2007 by the Netherlands Cancer Society. Its aim was to assess the quality of cancer care and develop strategies for improvement, and the main objectives included analysis of the relationship between the number of surgical procedures (high-volume centers) and outcomes, as well as identifying factors that influence quality of care differences by region and hospital type. The program revealed that adherence to guidelines varies across hospitals, e.g., the percentage of patients who had at least 10 lymph nodes assessed during colorectal cancer surgery ranged from 20% to over 70% across facilities. Furthermore, the program confirmed that centralizing high-risk and more complex procedures around high-volume centers contributed to lower mortality rates. Thus, in accordance with its authors’ assumptions, the program confirmed the optimal direction of actions aimed at concentrating complex medical procedures in specialized centers and the need for ongoing monitoring of results, as well as the importance of making them available to appropriate specialists in order to further improve quality of care [62].

The vital role of monitoring was also confirmed in another study focusing on quality monitoring using indicators. In the 2013 study by Winters-Van Der Meer et al., the authors focused on examining the quality of long-term care in the Netherlands between 2007 and 2009 based on QIs, taking regional perspectives into account. An improvement was observed in the indicators concerning “somatic care” and “home care”. In the case of “somatic care”, 9 out of 15 indicators improved, including assessment and evaluation of the care plan and patient participation in decision-making, each of which increased by about 7–8%. Similar results were shown for “psycho-geriatric care” with regard to pressure ulcer rates (23% decrease), medication-related incidents, and depression (10% decrease). Nevertheless, some deterioration was also shown in 8 of 15 indicators related to comfort and atmosphere in long-term care facilities. The study also showed differences in the results achieved between regions. Nonetheless, the authors concluded that the use of indicators in long-term care can help to improve results [63].

Another initiative that may indicate the beneficial impact of monitoring and using indicators for quality improvement is the 2009 initiative by the Dutch Institute for Clinical Auditing (DICA) to integrate nationwide medical outcome registries to improve the quality of healthcare and reduce treatment costs. The initiative included more than 19 registries for various conditions, including colorectal cancer, breast cancer, Parkinson’s disease, etc. DICA supports data collection and analysis, involving clinical specialists, service providers, and fund holders. In the report published by DICA, “Building National Outcomes Registries in the Netherlands”, significant changes were identified that suggested improved clinical outcomes. Amongst others, between 2009 and 2014, the complication rate for colon cancer surgery decreased from 33% to 30% and for rectal cancer from 40% to 37%. Furthermore, the re-intervention rate decreased from 17% to 13% and the in-hospital mortality rate from 5.8% to 2.7% for colon cancer and from 3.8% to 1.1% for rectal cancer. Since 2011, the breast cancer registry has shown improved treatment rates, e.g., a reduction in the percentage of tumor-positive margins (indicating the presence of cancer cells after surgery) from 6.1% to 4.6% for invasive breast cancer. Importantly, the analysis of data for colon cancer in 2011–2012 showed that complications increased the costs of care by 26% for mild complications and by 196% for severe complications. Due to the decrease in the number of complications following the implementation of DICA guidelines the costs of treatment in hospitals was reduced by a significant amount, which may provide a substantive and economic basis for further monitoring of results [64].

The Netherlands Cancer Registry (NCR) is the source of comprehensive cancer data in the Netherlands. The NCR is a national cancer registry system that has been operating and collecting data since 1989 and is managed by the Netherlands Comprehensive Cancer Organisation (Integraal Kankercentrum Nederland (IKNL)). The registry collects and provides data on cancer-related morbidity, survival, and mortality. Its database includes all cancer cases diagnosed in Dutch hospitals and, thanks to data registration by specialists at the IKNL, ensures that the data are reliable and objective. The NCR contains detailed information about patients, including TNM stage, tumor location, morphology, comorbidities at diagnosis, and treatment received immediately after diagnosis. The NCR aims to provide comprehensive cancer data for use in epidemiological research, clinical research support, clinical practice assessment, policy development and updating, screening program evaluation, international comparisons of morbidity and survival, health policy formulation, and analysis of cancer incidence in specific populations. Furthermore, to enable more detailed research on the diagnostic pathway and management of cancer care in primary care, the NCR has been linked to the General Practitioner (GP) database within the PHARMO Database Network. This has created the NCR-PHARMO GP Cohort, which includes 135,868 patients diagnosed between 2006 and 2014, providing additional information on the use of primary care before and after cancer diagnosis, medications prescribed, laboratory test results, and referrals to specialists [65,66].

The OECD 2023 report presenting the Netherlands Cancer Profile showed that five-year survival rates for cancer had improved over the previous decade. As the authors of the document pointed out, this was partly due to a strong cancer care network, which was supported by continuous improvements in diagnostic and treatment procedures, a comprehensive national cancer registry, and a strong clinical audit system. The data presented in the report indicated that cancer mortality decreased significantly between 2011 and 2019, while the burden of cancer increased (probably as a result of better detection methods and an aging population). Despite the fact that the overall incidence of cancer was higher than the European Union (EU) average, the significant improvement in cancer mortality rates over this period, which showed a decrease of 11%, placed the Netherlands among the EU countries with the largest reduction in cancer mortality. Age-standardized cancer mortality decreased for most major cancer types, with the exception of esophageal cancer, which increased slightly (4%) over the reported period. The largest percentage changes in cancer mortality during this period occurred for stomach, colon, breast, and lung cancers, ranging from an 18% decrease for lung and breast cancer to a 27% decrease for stomach cancer. Between 2000 and 2018, the potential years of life lost due to malignant neoplasms decreased by 30%, representing 1249 years of life lost among 100,000 people aged up to 75 years in 2018. The relative decrease was larger among men (34%) than women (25%), with 2256 and 1244 years of life lost, respectively, in 2018. As mentioned earlier, the Netherlands has a sophisticated clinical audit system. Over the years, national quality guidelines for cancer care (including rare cancers) have been introduced, supported by an audit system run by the non-profit Dutch Institute for Clinical Auditing. The Netherlands also has several registries that monitor different indicators for multiple cancer types (mostly surgically based). In addition to measuring clinical outcomes, some registries also use PROMs to support a process of improving quality of care that takes into account the patient’s perspective. The audit system covers breast, colorectal, gynecological, head and neck, liver, lung, pancreatic, skin, and upper gastrointestinal cancers. A prostate cancer registry has also been operational since the end of 2022. These registries are being implemented nationwide, providing participating healthcare providers with access to the performance indicators they develop through reports and a dedicated dashboard, ensuring transparency of quality-of-care data for all stakeholders in the healthcare system [67].

### 3.6. Germany

**Figure 5:**
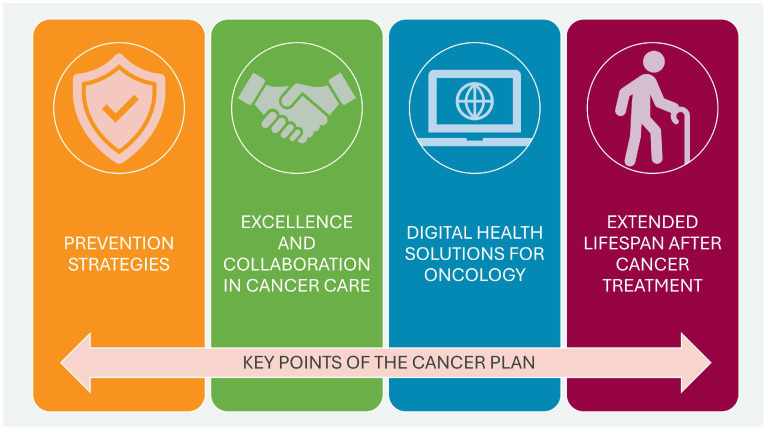
Key points of the German National Cancer Plan. Source Nationaler Krebsplan Handlungsfelder, Ziele, Umsetzungsempfehlungen und ErgebnisseBundesministerium für Gesundheit [70].

In June 2008, the National Cancer Plan (der Nationale Krebsplan (NKP)) was initiated as a program for coordination and cooperation, aimed at further developing and improving cancer care and early detection of cancer in Germany. In addition to the initiators, namely the Federal Ministry of Health, German Cancer Society, German Cancer Foundation, and Association of German Cancer Centers (Bundesministerium für Gesundheit, Deutsche Krebsgesellschaft, Deutsche Krebshilfe und Arbeitsgemeinschaft Deutscher Tumorzentren), more than 20 organizations and 100 experts participated in the program. In the years 2008–2011, 13 goals were formulated in four priority areas of action, together with some 40 subgoals and around 100 recommendations for implementation being defined. The main task of the NKP was to connect decision-makers and experts from institutions and organizations involved in oncology in order to focus efforts on the fight against cancer and achieve concrete results. In 2024, the Federal Ministry of Health took over responsibility for reorganizing the plan, and its activities focused on four main areas (see Figure 5). The National Cancer Plan was, among others, the basis for the creation of an Act of Law on the “early detection and registration of cancer”. This Act created a framework for significant changes in the field of early cancer detection [68,69]. In the second area of activity, which was defined as “further development of oncological care structures and quality assurance”, goals 4 to 9 were formulated (see below), which focused on improving the quality of oncological care. Within the framework of goal 8, the focus was on reliable reporting of quality in oncology for service providers, decision-makers, and patients through:

1. Widespread development of clinical cancer registries to monitor the quality of care for all cancer patients;
2. Strengthening the cooperation of regional clinical cancer registries;
3. Ensuring strong collaboration between clinical and epidemiological cancer registries;
4. Providing feedback to all service providers involved in the form of a structured, critical assessment of performance;
5. Transparent presentation of care outcomes to all stakeholders, including clinics, physicians, patients, and the public;
6. Introduction of uniform standards for documentation of cancer data.

A milestone in the implementation of health policy was further legislation in the form of the Act on the “further development of early cancer detection and quality assurance”, which came into force on 9 April 2013. The Federal Cancer Early Detection and Registration Act (Krebsfrüherkennungs und Regi Stergesetz—KFRG) implemented the recommendations of goal 8 (above) and created the necessary legal framework for the general introduction of clinical cancer registers in Germany. The individual states are tasked with establishing registers that meet the requirements of § 65c SGB V Social Security Code (Sozialgesetzbuch (SGB) Fünftes Buch V). This includes collecting all data on the occurrence, treatment, and course of oncological diseases, analyzing them, and making the results of the analyses available to the doctors involved in the treatment process. In order to ensure the most consistent process possible, the states have established a special working group for the implementation of the KFRG. The Federal Ministry of Health (BMG) supports these activities [69,70]. There are now also nationwide institutions, such as the German Childhood Cancer Registry (GCCR), which has been collecting data on cancer in patients under 18 years of age since 1980, and the German Centre for Cancer Registry Data at the Robert Koch Institute, which collects data from national registries in order to create a nationwide database [71].

In 2008, the Association of Scientific Medical Societies in Germany, the German Oncology Society, and the German Oncology Aid (der Deutschen Krebsgesellschaft, der Arbeitsgemeinschaft Wissenschaftlicher Medizinischer Fachgesellschaften und der Deutschen Krebshilfe) jointly launched German Oncology Guideline Program (GGPO). The program aimed to support the development, implementation, and evaluation of evidence-based clinical practice guidelines (CPGs) in oncology, in line with the goals of the German National Cancer Plan [72]. A mandatory part of any GPPO CPG development process is the definition of QIs based on strong recommendations. These recommendations are considered appropriate as a quality standard because the actions they address can be assumed to be beneficial for most patients. The indicators are selected in a well-defined, multi-step process by multidisciplinary experts from the guideline development group. The basic criteria for selecting QIs include the strength of the underlying recommendation (strong, grade A), the potential for improving care, and measurability. The QIs derived from the CPG are implemented in cancer centers certified by the European Cancer Centers (ECC). All indicators must be reported annually by each certified center. The speed of implementation of the indicators, and thus adherence to the guidelines, is monitored and assessed via the ECC certification system. In addition, the results of the QI indicators are regularly communicated to the GGPO guideline groups to ensure the best possible exchange between guideline development and routine clinical practice. In the context of the guideline update, existing QIs are also subject to an update process. As of January 2022, 31 GPPO guidelines have been published for both specific tumors and cross-sectional health issues and 192 QIs have been derived from them. Of these, 108 QIs are being implemented in 18 tumor-specific certification procedures in a total of 1988 certified centers [73].

Clearly, a fundamental expectation of ECC certification is that centers that have achieved this recognition should demonstrate better treatment outcomes in their patients compared to those treated in non-certified centers. A retrospective cohort study by Bierbaum, Bobeth, and Roessler et al. [74] covering the years 2009–2017 aimed at addressing this question, using patient data containing demographic information, diagnoses, and treatment methods that were available from national health insurance registers in relation to the treatment center’s status with regard to certification. The aim of the study was to determine whether patients with incidental colorectal cancer or rectal cancer who had received treatment at a certified oncology center demonstrated better treatment outcomes. It was ensured that important methodological considerations were met, including the use of relatively large patient samples, namely colon and rectal cancer cohorts consisting of 109,518 and 51,417 patients, respectively, who had been treated in a total of 1052 hospitals. Relative survival analysis was used in analyzing the results, taking into account mortality data from the German population and adjusting for patient and hospital characteristics using Cox regression for patients in certified and non-certified hospitals. A total of 37.2% of patients with colon cancer and 42.9% of patients with rectal cancer were treated at a certified center. Patient age, gender, comorbidities, secondary malignancies, and distant metastases were similar across groups (certified/noncertified) for both colon and rectal cancer. However, relative survival analysis demonstrated that patients with colon cancer treated at a certified center showed significantly greater survival rates of 68.3% at 5 years, compared to 65.8% of patients treated at noncertified hospitals. Similarly, the 5-year survival rates for rectal cancer treated at certified centers was 65.0%, compared to 58.8% at non-certified centers. Cox regression with appropriate coefficients showed a lower risk of death for patients treated in certified centers for both colorectal and rectal cancer.

### 3.7. France

In France, the fight against cancer has been based on national plans since 2003, aimed at mobilizing public health facilities around prevention, screening, organization of care, and support for patients and their carers both during the course of the disease and after treatment. Since 2003, three cancer plans have been established in succession, up to 2019. France is currently implementing a 10-year cancer strategy for 2021–2030. The implementation of the mission defined in the ten-year plan was entrusted by the law of 8 March 2019 to the National Institute of Oncology (Institut National du Cancer) [75].

The National Cancer Plan 2003–2007 was the first phase of a strategic program for a five-year period. It covered six priority areas: prevention, screening, treatment, support, research, understanding, and discovery, in order to achieve the main objective of the plan, which was to reduce cancer mortality by 20% over five years. The plan also set out key quantitative indicators representing the individual objectives, namely:

- Prevention—To reduce smoking among youth by 30% and among adults by 20%, and to reduce the number of adults addicted to alcohol by 20%.
- Screening—Implementation of consistent screening strategies across the country. For breast cancer, 80% of women aged 50 to 74 should benefit from screening. For cervical cancer, a target of 80% of women aged 25 to 69 was set. For colorectal cancer, a pilot screening program was initiated, which could be extended to the general population in the future.
- Treatment—Providing all patients with a personalized care program and access to multidisciplinary care within an organized healthcare network.
- Support—Ensuring patients have the right to obtain high-quality information about medical facilities providing treatment for cancer in their region as well as improving the way diagnosis is communicated and providing better psychological support to all patients.
- Research—The priority was to create a nationwide cancer monitoring system. In particular, the goal was to include at least 10% of patients in clinical trials at reference centers and the development of large-scale genomic cancer programs (the aim being to create a database of 100,000 cancer samples for clinical–biological analysis).

The Cancer Action Plan included a package of 70 different measures/actions that required the creation of a dedicated coordinating mechanism [76,77].

In addition, the first plan included the establishment of a National Cancer Institute (Institut National du Cancer) as a key to better coordination of all bodies involved in the fight against cancer. The National Cancer Institute was established under the Public Health Act of 9 August 2004. The intention was that the Institute should closely involve both the medical community and patient representatives in its activities to facilitate the implementation of the cancer plan. The Institute was to provide a national vision for the fight against cancer, from tracking epidemiology to monitoring threats to care networks and facilities. The Institute was to be a vigilant participant in the introduction of the oncology plan and was expected to stimulate and monitor the implementation of provisions regarding the quality and coordination of care, as well as to promote equal access of patients to the best care, regardless of their place of residence [76,77].

The next plan, adopted for the period 2009–2013, was a continuation of the 2003–2007 Cancer Plan and, to some extent, built on the assumptions set out in the earlier document. However, considerable consolidation work was necessary in some cases to ensure implementation of the actions and in others, to adapt the way in which they were introduced and carried out. As a result, new proposals were put forward to give impetus to the new objectives, with particular emphasis on new research and innovation efforts. These included the transfer of such innovations to the healthcare system; improved consideration of health inequalities in relation to cancer and implementation of actions to correct them; better coordination of patient care, including the extension of care beyond the hospital environment through more effective involvement of referring physicians; and new medical and social initiatives to provide better support for people living with and after cancer. The plan adopted 5 main areas of action, 30 measures, and 118 actions [78]. The document included six flagship measures assigned to the areas of action, and these measures are presented in Table 3.

According to the data included in the final report on the implementation of the plan (Cancer Plan 2009–2013 Rapport Final Au Président de La République Juin 2013), most of the activities planned for the years 2009–2013 had been implemented [79].

The basic assumption of the Cancer Plan for 2014–2019 was to meet the needs and expectations of sick people, their loved ones, and all citizens. As part of the third edition, four main areas of action (ambitions) were adopted, and each of them was assigned specific goals and actions aimed at achieving the prioritized goals [80]. Table 4 presents a synthetic summary of the plan.

Under goal 2, a number of actions were adopted related to the development of QIs, both in the area of waiting times for healthcare services and regarding clinical practice itself. It was also assumed that the results would be gradually made public in order to better inform patients and ensure transparency in the field of quality of care [80].

In 2019, a report was published for the first time with a proposal for quality and safety of care (Indicateur de qualité et de sécurité des soins) indicators specific to breast cancer. These indicators were based on good practice recommendations and could be automated via medical–administrative databases (activity 2.2 of the Cancer Plan 2014–2019). For each indicator, detailed definitions, calculation rules (numerator and denominator and procedure codes to be included in the measurement process were developed), indicator type, inclusion and exclusion criteria, geographic scope, measurement frequency, explanation of the indicator rationale, and target (expected) and alert values were developed [81].

In July 2020, the report “Evaluation of the Third Anti-Cancer Plan 2014–2019” (Evaluation du troisième Plan cancer 2014–2019) was published. The report described the importance of the actions carried out under the third cancer plan, but also indicated areas in which specific goals had not been achieved. Many bodies dealing with cancer issues had experienced “fatigue” with the planned approach. Additionally, the report indicated difficulties in monitoring the plan (17 objectives, 208 actions, over 1500 intermediate steps), concluding that the multitude of actions had significantly hindered the clear identification of priorities. Additionally, it emphasized the poor identification of the specificity of the third cancer plan compared to previous plans, and the lack of strategic coordination of the activities of different entities. Since the first cancer plan in 2003, the number of public health programs had increased significantly, creating many intersections that raised questions concerning coordination and effectiveness. Another limitation of the cancer plan identified by the report was its duration. The authors of the report felt that the five-year plan did not allow for the assessment of the impact of the actions taken in many areas (e.g., prevention, research). This also raised the question of responsibility for the results obtained. It was suggested that extending the duration of the strategy to ten years, as provided for in the current law, might be a more appropriate format for achieving the intended objectives, if the programming and strategic directions were regularly reviewed [82].

In a study by Houzard, Courtois, Le Bihan, et al. [83], “Monitoring breast cancer care quality at national and local level using the French National Cancer Cohort”, ten QIs were selected and analyzed. The authors showed that in France, the care of patients with breast cancer (BC) was in compliance with most of the QIs with regard to the percentage of patients who underwent biopsy before the first treatment (94.5%), the percentage of patients who underwent adjuvant radiotherapy after breast-conserving surgery (94.5%), the percentage of women who underwent radiotherapy within 12 weeks after surgery without chemotherapy (86.2%), the percentage of patients with ductal carcinoma in situ (DCIS) who underwent immediate breast reconstruction (54.3%), and the percentage of women with breast cancer in situ who underwent surgical reintervention (14.4%). The authors emphasized that some indicators were still far below the recommended level. They also noted that some QIs differed significantly depending on the region or patient specificity.

Cancer registration in France takes place at the level of the administrative territorial units of the country (departments). However, currently, the key role in monitoring, researching, and supporting the fight against cancer in France is played by the Réseau Français des Registres des Cancers (FRANCIM). This is a network of registries that, in cooperation with the Comité d’Évaluation des Registres, collects and analyses data from the main cancer registries in France. According to the information posted on the FRANCIM website, in 2023 the network included 30 cancer registries that monitored all types of cancers in adults in 22 departments and specific cancers in 6 departments. Additionally, the network registers cases of cancer in children throughout the country. The main goal of the network is to provide the necessary data for making decisions in the area of public health and to enable comprehensive epidemiological studies [84]. Due to the fact that the operating registries cover about 24% of the French population, in April 2023, a bill to establish a national cancer registry was submitted to the Senate, the implementation of which would be the responsibility of INCa. According to the explanatory memorandum, the national registry would not replace existing registries; rather, its task would be to expand the process of registering new cancer cases in order to precisely determine national incidence and mortality rates. In addition, according to the authors of the draft law, the registry would follow the recommendations of the IARC and the European Committee for Cancer Research, in terms of the minimum set of data collected, for each type of cancer. The introduction of consistent sampling rules would be applied that would help to avoid duplication of tests and enable epidemiological monitoring and tracking of the patient’s pathway [85].

### 3.8. Italy

Italy’s early efforts towards a national oncology plan date back to the late 1990s, when the National Health Plan for 1998–2000 (Piano Sanitario Nazionale 1998–2000) formulated strategic objectives for the prevention, diagnosis, and treatment of cancer, which were consistently included in the successive editions of the National Health Plan [86,87]. However, the key document introducing significant organizational changes in the Italian healthcare system aimed at combating cancer appears to have been the National Oncology Plan for 2011–2013 outlined in the document entitled “Technical policy document on the reduction of cancer disease burden for the years 2010–2013” (Documento Tecnico di Indirizzo per Ridurre il Carico di Malattia del Cancro Anni 2011–2013). This plan was developed by the Italian Ministry of Health and published in 2011. It defined strategic goals in the field of prevention, diagnosis, and treatment of cancer and envisaged the creation of an integrated oncology network at the national level (Rete Oncologica Nazionale). Moreover, the plan stressed the need for coherent cooperation between the regions and the central government and took into account the challenges related to the aging population and the rising costs of healthcare. Its implementation aimed to achieve sustainable, high-quality oncological care accessible to all Italian citizens. In addition to such goals as reducing the burden of cancer by reducing mortality and ensuring equity in access to diagnostics and treatment between the regions, there were also goals such as improving the quality of care by implementing uniform standards and tools enabling management on a national scale. The emphasis on improving quality was also expressed through the need to develop indicators for assessing the processes and outcomes of treatment. The plan also placed great emphasis on expanding screening programs for cervical, breast, and colon cancer, as well as implementing systems to monitor the quality and effectiveness of these programs. Key actions also included campaigns promoting a healthy lifestyle, HPV vaccinations, and the development of palliative care, with the participation of non-governmental organizations, supporting research, and the implementation of innovative solutions in treatment. The operational objective of the plan was, among others, to increase the registration of cases of cancer from 32% to at least 50% of the population affected by the disease [88]. Table 5 summarizes the main priorities of the Italian National Oncology Plan 2023–2027.

The Italian healthcare system has independently implemented advanced quality and outcome monitoring tools, which have been crucial for assessing the effectiveness of health policies. In the field of oncology, QIs and cancer registries play a particularly important role, providing data on disease incidence and effectiveness of interventions. The Griglia LEA system monitors the quality of care at the regional level, and includes indicators on participation in screening tests, such as mammography or colorectal cancer screening, as well as cancer treatment outcomes. The National Outcomes Program (Programma National Esiti) tracks 129 indicators, including 30-day mortality after cancer surgery (colon, lung, and stomach cancer), readmissions, and outcomes of surgical interventions. Data collected at the hospital and regional levels allow for the assessment of local differences in treatment outcomes. Additionally, the hospital discharge database (Scheda di Dimissione Ospedaliera) collects detailed data on patients hospitalized for cancer, including diagnoses, medical procedures, and treatment outcomes. This information allows monitoring of the burden on the healthcare system and assessing the quality of care in different facilities [90].

An important and independent complement to these activities is the national cancer registry (Registri Tumori), an indispensable tool in the fight against cancer supporting both research and practical actions at the local and national levels. The activities of the cancer registries in Italy have been coordinated by the Italian Association of Cancer Registries (Associazione Italiana registri tumori) since 1996. Currently, the network includes 50 population registries and 7 specialist ones, monitoring 70% of the country’s population, which, interestingly, is in keeping with the operational goals of the program from 2011–2013, assuming the completeness of cancer registration at the level of at least 50%. Data from the registries cover over 3 million people diagnosed with cancer, which include about one million deaths [89].

An example of an advanced regional approach is the Tuscan Performance Assessment System, which comprises over 130 indicators, including those related to the quality of cancer treatment. As indicated by the authors of the report “OECD Reviews of Health Care Quality: Italy, Raising Standards”, this system significantly improved treatment outcomes in the region between 2006 and 2010 (with around 50% of indicators demonstrating a significant improvement in annual results), becoming an important driver for other Italian regions to take similar measures. One of the positive results achieved by the constant updating and monitoring of oncological epidemiology, which allows for the assessment of the impact of cancer prevention strategies and diagnostic and therapeutic systems, is the five-year survival rate. According to the report “I numeri del cancro in Italia” for 2019, people who became ill between 2005 and 2009 showed higher five-year survival rates than those who became ill in the previous five years, in the case of both men (54% vs. 51%) and women (63% vs. 60%). It is worth noting that these small differences in percentage values translate into significant improvements for several thousand people compared to previous years. Moreover, according to the report, there are different trends in cancer incidence by region, gender, and type of cancer. In men, the largest decrease was observed in northern regions such as Emilia-Romagna and Trentino (2.3% and 2% per year, respectively), while in central and southern regions, the decreases were smaller or the incidence remained stable. In women, the decrease was less significant; for example, in South Tyrol (0.6%) and Lombardy (0.5%). Breast cancer showed a small nationwide increase, especially in northern and central regions, which may have been due to the expansion of screening, with southern regions seeing more significant increases. For prostate cancer, there was a nationwide decrease, which may be related to the lower use of PSA testing, especially in northern regions such as Friuli and Liguria (8.3% and 4%, respectively). Colorectal cancer demonstrated a decrease in incidence in both men (1.3%) and women (1.1%). Lung cancer in men showed a decreasing trend (1.6%), while in women an increase was observed almost everywhere in Italy (with the highest values increasing by 3.2%). As regards the general trend of mortality from cancer, a decrease was recorded in both men (0.9%) and women (0.5%), although the rate of change varied according to the type of cancer and the region. Mortality from stomach cancer decreased significantly (2.4% in men and 2.7% in women), except in the southern regions, where it remained stable in men. A similar decrease was observed for colorectal cancer (0.7% in men and 0.9% in women). In the case of lung cancer, the rate decreased in men (1.6%), especially in the central and northern regions, while in women it increased (1%) in all regions of the country. As regards breast cancer, mortality among women decreased (0.6%), and a significant decrease (1.9%) was also observed for prostate cancer [91].

### 3.9. Spain

The main initiative in Spain, although preceded by many less centralized actions to combat cancer, was the strategy entitled “Estrategia en Cáncer del Sistema Nacional de Salud”. The plan, developed by the Spanish Ministry of Health, dates back to 2003, but the strategy itself was approved by the Interterritorial Council of the National Health System (Consejo Interterritorial System Nacional de Salud) in 2006. Its creation was a response to the growing health challenges related to cancer, which was one of the main causes of death in Spain. The preparation of the strategy was the result of extensive cooperation between medical experts, scientists, patient organizations, and representatives of the autonomous communities (Comunidades Autónomas). The key objective was to coordinate activities at the national level in the field of prevention, diagnosis, treatment, and research on cancer. The strategy was part of the National Quality Plan (Plan de Calidad para el Sistema Nacional de Salud) and aimed to harmonize activities at the national level in order to ensure equal access to effective cancer treatment and prevention methods. The strategy was to be evaluated in two-year cycles, and its implementation was to be monitored through QIs and mechanisms for assessing effectiveness. The strategy set several key objectives. In the area of health promotion and prevention, the focus was on educational activities promoting a healthy lifestyle, such as reducing tobacco smoking, combating obesity, and promoting physical activity. These campaigns aimed to reduce exposure to cancer risk factors. Early detection included the development of screening programs for breast, cervical, and colon cancer and setting standards for population-based screening programs to increase the detection of cancers in the early stages. In the field of treatment, the creation of multidisciplinary treatment teams that could provide comprehensive oncological care was promoted. Actions were also taken to improve access to palliative care and to provide psychological and rehabilitation support for patients. Another objective was to increase support for epidemiological, clinical, and basic research in order to develop new diagnostic and therapeutic methods. A separate but particularly important point included in the strategy were population-based cancer registries (developed since the 1960s and operating in many provinces), which played a key role in the development of oncological strategies, especially in monitoring cancer-related morbidity and mortality. A number of indicators were used to assess progress in implementing the strategy, such as the percentage of the population covered by screening programs and reduction in mortality due to the most common cancers, such as lung, breast, and colon cancer. The first evaluation of the strategy took place in 2008, providing key data on progress in achieving its goals, such as reducing tobacco smoking and implementing screening tests. As a result of this evaluation, the strategy was updated in 2009, taking into account the latest scientific data and the health situation in the country. Subsequent evaluations were planned in two- and four-year cycles, which was to enable monitoring of progress and adjustment of activities to changing needs. When updating the strategy, QIs were also redefined and adjusted to reflect the current state of knowledge and experience [92,93]. A summary of the main priorities of the Cancer Strategy of the National Health System in Spain is presented in Table 6.

The report “Evaluación de la práctica asistencial oncológica” presented the results of the activities carried out in attempting to meet the efforts and assumptions laid out in the document “Estrategia en Cáncer del Sistema Nacional de Salud”. As the authors of the report pointed out, key structural indicators such as the presence of oncology committees (de comité de cáncer, tumor committee(s)), clinical protocols, and access to psychological support were generally met in most hospitals. Oncology committees were present in most facilities, although their presence was less common in the treatment of colorectal and lung cancer than in the treatment of breast cancer. In small hospitals, alternative solutions such as general tumor committees were often implemented, but their composition was not always optimal (due to a lack of radiotherapists). Clinical protocols were widely available but not always adapted to local needs, and in some facilities, formal measures requiring their use were inadequate. Moreover, psychological support for patients was considered to be insufficient in practice, and in many cases relied on external resources, which indicated the need for greater integration of this element into treatment plans. The analysis of process and outcome indicators showed mixed results. In the case of breast cancer, only 60% of cases came to the attention of the oncology committees (which was probably due to time limitations). Preoperative TNM staging (realización del TNM) was low (25%) compared to postoperative (59%). Surgical procedures, such as sentinel node biopsy and removal of an appropriate number of lymph nodes, reached satisfactory levels (86% and 72%), in keeping with the strategy’s recommendations, and the rate of hormonal treatment for patients with positive receptors (receptores positivos) was as high as 90%. The indicators regarding delays in starting treatment were worthy of note, as only 44% of patients started therapy within the recommended time of 28 days, while for adjuvant treatment the rate was 66% for the appropriate time frame. The results also indicated that for colorectal cancer, there was an improvement in the number of cases discussed by the committees (42%), but this rate was still lower than for breast cancer. Medical records, especially pathology reports, often did not contain key information such as the status of surgical margins and the absence of mesorectal involvement (40%). According to the data, stoma care was available for only 49% of patients with a stoma. Furthermore, the rate of postoperative infection at the surgical site was quite high, at 20%, and was significantly higher than accepted national and international standards [95].

Reports from recent years, including “*Estrategia en Cáncer del Sistema Nacional de Salud*”, approved by the Interterritorial Council of the National Health System in 2021, indicate that between 2009 and 2018, cancer-related mortality decreased on average by 1.6% per year in men (mainly lung, prostate, and bladder cancer) and by 0.7% in women (mainly breast, colon, and stomach cancer). However, lung cancer remains the leading cause of death in men (although the mortality rate has decreased by 1.9% per year). In contrast, breast cancer remains the leading cause of death in women (although the mortality rate has decreased by 1.3% per year). Although it is not possible to clearly indicate the cause of the changes in these indicators, these data may indicate a positive effect of all the preventive, diagnostic, and subsequent interventions in a significant proportion of cancers [94].

### 3.10. Sweden

On 5 July 2007, the Swedish Government decided to establish a Commission of Inquiry, with the task of presenting proposals for a national cancer strategy (A National Cancer Strategy for the Future). According to the Government, the national cancer strategy was intended to focus on future needs and challenges. It was to take a comprehensive perspective and include primary prevention, early detection, diagnosis, treatment, and palliative care, as well as improving and disseminating knowledge in these areas. Particular attention was to be paid to the patient’s perspective. Thus, in line with the activities of the Commission, since 2009 Sweden has had a national cancer strategy, which has become the basis for many efforts at various levels to improve the quality of cancer care. It was deemed that the strategy should contain clear goals to drive implementation and enable assessment of whether the intended effects had been achieved. Five overarching goals were proposed within the strategy. These were:

1st  Reducing the risk of cancer;
2nd  Improving the quality of treatment of oncology patients;
3rd  Extending survival time and improving quality of life following a cancer diagnosis;
4th  Reducing regional differences in survival after a cancer diagnosis;
5th  Reducing differences in morbidity and survival between population groups.

In order to achieve the goals set out in the strategy, a number of actions were planned, according to the areas concerned (see Table 7).

The National Board of Health and Welfare was tasked with establishing benchmarks for the above objectives, i.e., current levels and status as a basis for further monitoring. The committee proposed that the cancer strategy be established by agreement between the Government and the Swedish Association of Local Authorities and Regions. It was considered that consensus and cooperation at all levels were essential for effective implementation, in addition to a clear implementation structure and clearly formulated follow-up activities. The expectation was that follow-up activities should take place continuously and be integrated with the implementation and form the basis for regular revision of the strategy. One of the key objectives of the strategy was to strengthen the use of QIs to monitor and improve the effectiveness of cancer care, with a focus on patient-centered outcomes, early diagnosis, and adherence to clinical practice guidelines [96].

In 2018, the Government decided to provide a long-term orientation for oncological care. The long-term orientation indicated the Government’s priorities, but did not contain binding measures and was to be operationalized primarily through agreements and the assignment of powers to specific bodies.

In June 2023, the Authority for Care and Care Analysis (Myndigheten för vårdoch- omsorgsanalys) was commissioned by the Government to prepare an analysis of the implementation status of the national cancer strategy, taking into account the perspectives of the patient, healthcare provider, and system. The aim was to identify strengths and areas for improvement in the national cancer strategy. The analysis also included standardized treatment processes and care for children with cancer. In addition, the assignment included identifying external factors that might influence the need to modify the national cancer strategy. The report was published in 2024, and the picture outlined in it was built on the basis of three questions:

- What are the strengths and development needs in terms of management through an anticancer strategy?
- What are the strengths and development needs for achieving goals and implementing interventions within the oncology strategy?
- What environmental factors create the need for changes in the cancer strategy?

The main conclusions of the report were as follows:

- Although the national cancer strategy provides structure and direction for action, gaps were identified in terms of prioritization, implementation, and monitoring of progress.
- Differences were noted in the implementation of objectives and actions taken depending on the individual areas covered by the strategy.
- It was demonstrated that standardized treatment processes have created coherent care pathways for oncology patients, but an evaluation of their effectiveness and a review of activities related to reducing waiting times are required.
- Within the framework of care for children with cancer, areas requiring improvement were identified.
- It was recommended that when developing a new strategy, the development of medical technologies, EU initiatives, and the transition to a healthcare model based on high quality and accessibility of services should be taken into account [97].

When analyzing the assumptions of the national strategy for combating cancer, it is impossible not to mention the Swedish Cancer Registry (Cancerregistret), which was established in 1958 with the support of the King. From the very beginning, registration of cancer cases was managed centrally by the Medical Board, later the Board of Health and Welfare. In the mid-1970s, regional cancer registries were established in the country, which took over the registration. Since the early 1980s, there have been six oncology centers, later regional oncology centers, to which reports are sent for coding and registration. After checking the material, it is sent annually to the National Board of Health and Welfare for inclusion in the Cancer Registry [98]. There are also National Quality Registries in Sweden, which are intended to facilitate monitoring and evaluation of the results and quality of healthcare. The National Quality Register in the field of oncology contains individual data on diagnoses, treatments, and outcomes. The registry enables tracking of healthcare activities for all patients in the country within a specific diagnostic area. Quality registries also provide a basis for monitoring regional activities and research. In Sweden, there are 30 national cancer registries, managed by six regional cancer centers. They currently cover the entire country and have been using a common platform (INCA—Information Network for Cancer) since 2008. Compared to the Swedish Cancer Registry, they offer a higher level of detail, including diagnostics, tumor characteristics, and treatment, with a rate of complete data in excess of 95% [98,99].

Data from the registers are used by the Swedish Government to assess quality of care using QIs. Since 2006, the Swedish National Board of Health and Welfare and the Swedish Association of Local Authorities and Regions have published reports entitled “Quality and Efficiency in Swedish Health Care—Regional Comparisons”. In 2011, the first report was published, presenting a comparison that reflected the quality of care in the case of the ten most common types of cancer, entitled “Quality and Efficiency in Swedish Cancer Care: Regional Comparisons 2011”. This report compared different counties in terms of treatment outcomes, patient experiences, and waiting times. The report stated that cancer patient survival rates were increasing, i.e., the relative five-year survival rate for men increased from just over 50% in 1990–1994 to almost 70% in 2005–2009. For women, the survival rate increased from 60% to 68%. The frequency of multidisciplinary team meetings and waiting times varied between counties. In addition, there was an improvement in the diagnostic efficiency of some cancers, e.g., a reduction in the number of bone scans for low-risk prostate cancer; for kidney cancer, 83% of patients had a CT scan, which is close to the target rate; and for lung cancer, almost all counties met the biopsy requirement at a level of 99% of patients [100].

Cancer registries are an excellent tool for improving outcomes for cancer patients. For example, according to data presented in a report by the Boston Consulting Group, one area in which the Swedish cancer registries have dramatically improved treatment and outcomes is acute lymphoblastic leukemia (ALL). Children treated for ALL were monitored by different cancer registries, which provided pediatric oncologists with detailed information on the characteristics of treatment and outcomes of individual patients. Based on these data, pediatric oncologists were able to both improve ALL treatment by introducing new drugs and optimizing treatment doses, and adapt ALL treatment to subpopulations of patients with different treatment responses. These modifications increased the five-year survival rate for ALL in Sweden from 12% (1968–1972) to 89% (2002–2005) [101].

Another example of the positive impact of registries on patient outcomes is the Swedish National Prostate Cancer Registry (The National Prostate Cancer Register, NPCR), which publishes annual reports on the quality of care and outcomes of patients with prostate cancer. Data from the registry have led to increased use of clinical guidelines and changes in preoperative diagnostics for patients with low-risk prostate cancer with the introduction of the PSA test as a screening tool, which has reduced the need for extensive imaging. Medical societies recommend limiting the use of bone scans, which rarely show metastases in low-risk patients and more often give false positive results. The NPCR monitors the proportion of patients undergoing bone scans to reduce unnecessary testing. A study by Cazzaniga, Ventimiglia, Alfano et al. [102] showed a decrease in the rate of such scans from 45% in 1998 to 3% in 2009. In addition, the authors also noted an increase in the use of local therapy for locally advanced prostate cancer. Among healthy men aged 70–80 years with high-risk nonmetastatic prostate cancer (defined as prostate cancer with no evidence of metastasis (N0 or Nx, M0 or Mx) and at least one of the following three criteria: Gleason score 8–10, local clinical stage T3, or prostate-specific antigen (PSA) 20–49 ng /mL), there was a significant increase in the use of treatment with curative intent, from 10% in 2001 to almost 50% in 2012. Another factor to which the authors drew attention was the increase in the use of active surveillance for low-risk prostate cancer, which was embodied in Swedish guidelines in 2007. Both this recommendation and the ongoing feedback from the NPCR to individual departments on compliance with the guidelines have potentially contributed to the increased use of active surveillance in Sweden. In fact, there has been a significant increase in the use of this treatment strategy among men with low-risk prostate cancer, from 40% in 2009 to 74% in 2014. Active surveillance was used even more frequently for very low-risk prostate cancer, from 57% in 2009 to 91% in 2014.

### 3.11. Denmark

The first cancer plan in Denmark was adopted by the Government in 2000. It focused mainly on epidemiological mapping of the incidence of different forms of cancer in Denmark compared to other Nordic countries. The mapping showed that Denmark significantly lagged behind its neighbors in promoting healthcare activities for dealing with cancer. The plan included activities to rectify these shortcomings and identify key areas for improvement in treatment practices. In addition, earlier proposals for the centralization of services were confirmed. This concerned, in particular, surgical treatment and radiotherapy, which were to be concentrated in larger hospitals to ensure that all patients had access to the best possible treatment and to enable more extensive use of expensive radiotherapy technology [103].

The document summarizing the activities carried out under the first cancer plan showed that Denmark had a higher cancer mortality rate than Norway, Sweden, and Finland. Analysis of data from the cancer registries in the countries concerned showed that Danish patients had a lower survival rate for four common cancers, which included lung cancer, breast cancer, rectal cancer, and colon cancer. The survival rate among Swedish patients was about 10% higher than for Danish patients, while the differences between Denmark, Norway, and Finland were generally less significant. The report emphasized that there was unlikely to be a single explanation for these differences, but potential causes included the higher consumption of calories, alcohol, and tobacco in Denmark compared to neighboring countries. In particular, Danish women smoke more than their Scandinavian counterparts, and such differences in lifestyle may explain why the incidence of cancer is higher in Denmark than in other Nordic countries. In addition, the report also presented recommendations and suggestions from the Cancer Steering Group on further work to improve treatment outcomes for Danish patients. Recommendations were presented for 10 areas: prevention, training of medical staff, contact and referral, organization of diagnostics and examination, expansion of diagnostics and treatment options, concentration of expertise in surgical treatment, screening, research and development, rehabilitation, and palliative care [104].

The second cancer plan, published in 2005, focused on improving the organization of patient flow in the treatment process, which later led to the development of cancer treatment packages. Focus was also placed on smoking prevention and strengthening oncological surgery. Two years later, the National Board of Health conducted a review of current treatment practices in Denmark. The review indicated the need for intensified efforts in the areas of care packages, oncological surgery, clinical guidelines, monitoring, consistency in patient care, and further actions to reduce tobacco consumption. After an intensive campaign by the Danish Cancer Society, the Government decided to adopt cancer treatment packages. These packages meant that hospital managers and relevant hospital departments had to increase their efforts to fully institutionalize optimal treatment procedures and, in particular, to reduce waiting times for diagnosis and treatment. These packages, combined with additional funding, were undoubtedly one of the most important improvements in cancer treatment in Denmark for several decades. In 2007, the parliament also adopted smoke-free regulations, which generally prohibited smoking in all public places and on public transport [103]. Among the recommendations made in the second cancer plan was the strengthening of the supervision and dissemination of data documenting the quality of oncological care services. In the field of oncology, considerable amounts of data were regularly collected and analyzed, but indicators suitable for regular monitoring of the quality of treatment had only been developed for a few areas. One of the general recommendations made in the second Cancer Plan was therefore the development of indicators for individual areas in order to enable the documentation of the quality of healthcare activities and the establishment of regular monitoring of these indicators. In addition, the plan recommended the establishment of centralized coordination and presentation of the collected oncological data [105].

The third cancer plan, adopted in 2010, included a parliamentary decision to strengthen cancer treatment, primarily by improving pre- and post-treatment activities. The aim was to accelerate the diagnosis of suspected cancer and strengthen early detection, and to improve post-treatment care, with a significant increase in rehabilitation and palliative care, which was to translate into an increase in the survival rate of cancer patients and improve their quality of life before, during, and after treatment [106]. While the previous cancer plan focused on increasing screening programs and streamlining and shortening diagnostic and surgical treatment, the third cancer plan took a more patient-centered approach, focusing on improving the quality of life of patients early in the treatment process and later. Therefore, in spring 2010, patients and their families were invited to a special reference group to provide input on the third cancer plan, which was implemented in late 2010 [103].

The fourth cancer plan, introduced in 2016, essentially sought to consolidate the previous plans by strengthening preventive measures and improving diagnostics and treatment flows. Also known as the Patient Plan, it essentially continued the principles of the previous plan, focusing on the patient’s quality of life and experience of the treatment process. This meant that only one doctor managed each patient, consulting with the patient and taking into account their interests when planning the treatment process. In addition, previous efforts were continued to further improve diagnostics and treatment with the intention of cure [103]. In January 2021, the last summary of the fourth cancer plan was published, which showed that all initiatives included in the fourth plan had been launched. Most of them were on track, some were completed, and some were delayed. For some initiatives, it was agreed by the parties involved in the implementation of the plan that modifications were required. It was agreed that actions would be taken to address delays in order to best support the objectives and goals. Plan four also included a number of initiatives that continued after 2020 [107].

Work is currently underway on the fifth edition of the plan, and funds for its implementation have been secured by the Danish Government as part of the “New Health Package” from May 2023. The Danish Health Authority (Sundhedsstyrelsen) is preparing the proposals for the fifth cancer plan, which will be presented to the Government at the end of 2024. Cancer plan five will be based on, among other things, early detection, improving care for patients after cancer treatment, and dealing with the late effects of the disease, rehabilitation, and palliative treatment [108]. A summary of the plans is presented in Table 8.

When discussing the quality of cancer care in Denmark, it is impossible not to mention the Danish Cancer Registry, which is the longest-operating registry in the Nordic countries. It was established as far back as 1942, and until 2013 it also collected data from Greenland. Until 1997, the Registry was run by the Danish Cancer Society. After cancer registration was transferred to the National Board of Health in 1997 (now the National Danish Health Data Authority), the Danish Cancer Society Research Centre received a copy of the datafiles for research purposes every year [98,109]. Denmark can be said to have made significant progress in developing the measurement of healthcare quality through clinical registers, although the hospital sector is better served by this initiative than primary and long-term care. At the end of 2010, the National Quality Improvement Program (Regionernes Kliniske Kvalitetsudviklingsprogram) was established to provide a framework for strengthening the infrastructure around clinical databases through the planned standardization of the operating conditions of some 60 national clinical registers [110]. As indicated by the WHO, Denmark is a pioneer in the field of digital health. At the individual level, people can access their electronic health records held by hospitals, municipalities, and family doctors. There are many examples of how data monitoring and analysis have helped to identify areas for improvement in the provision of health services and to develop the best solutions. One example of how data monitoring has led to observable improvements is the area of cancer survival. In the late 1990s, population data showed that five-year survival rates after a cancer diagnosis were significantly lower in Denmark than in neighboring European countries. This was followed by a concerted campaign that included restructuring cancer care services (centralizing surgical care to provide better-quality services in fewer hospitals), improving access to treatment, and implementing organized cancer screening programs. As a result, Denmark achieved an increase in survival from 48% (of people diagnosed with cancer and still alive after 5 years) in 2002 to 61% in 2014. Today, survival rates for various types of cancer in Denmark compare favorably with much of the EU [111].

As an example of how clinical practice can be improved, the Danish experience shows that a national quality management system, including national guidelines, a high-quality evidence base, frequent reporting, audits, and the involvement of all stakeholders, can contribute to improving outcomes and reducing regional differences. As described by Jakobsen, Green, Oesterlind, et al. [112], significant improvements were noted in all outcome measures over a 10-year period. These included an increase in the 1-year overall survival rate from 36.6% in 2003 to 42.7% in 2011, an improved 2-year survival rate from 19.8% to 24.3%, and, most strikingly, an increase in the 5-year survival rate from 9.8% to 12.1%. Moreover, the 5-year survival rate after surgical resection increased from 39.5% to 48.1%. Improvements were also noted in waiting times, concordance between cTNM and pTNM, and resection rates.

### 3.12. Norway

The first National Cancer Plan in Norway was developed for the years 1999–2003. The main goals of the plan were to reduce the number of new cancer cases through a long-term prevention strategy and to improve diagnostic and treatment services. The most important actions included:

1. Preventing new cases of cancer through actions aimed at reducing tobacco consumption, eating a healthier diet, increasing physical activity, and protecting against radiation.
2. Expanding the coverage of the breast cancer screening program to a national screening program for all women aged 50 to 69.
3. Increased radiotherapy capacity by adding 39 radiotherapy machines across the country.
4. Building competences in specialist health services—in particular, in the field of palliative treatment, hereditary cancers, and gene therapy.
5. Increasing the availability of key personnel, including medical specialists, oncology nurses, radiologists, and radiological diagnosticians.

The National Cancer Plan was assessed by the Foundation for Industrial and Technical Research in Norway, which is one of the largest European research institutes (Stiftelsen for industriell og teknisk forskning), and the Fafo Institute for Labor and Social Research (Forskningsstiftelsen Fafo) in spring 2004, and the results of this assessment were presented in the report “Implementation of the National Cancer Plan 1999–2003” (Iverksetting av Nasjonal Kreftplan 1999–2003). The general conclusion was that the plan should be implemented in accordance with the directives of the Norwegian Parliament. Since 2004, the activities resulting from the plan have been continued, as they are in essence in agreement with the aims of the new National Cancer Strategy. The first National Cancer Strategy in Norway was developed for the years 2006–2009 (extended to 2011). The strategy defined goals and activities at the national level in the areas of prevention, screening programs, diagnosis, treatment, and rehabilitation. It also defined goals in the areas related to the health workforce, registers, and research activities. The main goals of the cancer strategy are derived from the Soria Moria declaration (a Norwegian political statement forming the basis of Jens Stoltenberg’s second and first government) and are based on the general goals of health policy. The main areas of the strategy are prevention, national screening programs, patient-centered care, diagnostics, treatment, alternative treatments, rehabilitation, palliative treatment, personnel—capacities and competences, and the Norwegian Cancer Registry, National Quality Registries and Research [113].

In 2013, a new, comprehensive national strategy for combating cancer was developed for the years 2013–2017. In the strategy “Together Against Cancer” (Sammen—mot kreft), five main goals were defined:

- More patient-centered cancer care—This includes empowering patients to make informed decisions about their treatment, providing clear communication, and making sure patients know who to turn to for support at every stage of their disease.
- Norway as a leader in providing effective cancer care—This includes ensuring that at least 80% of patients receive treatment within 20 working days of referral. This was to be achieved through better coordination between providers and the establishment of specialist centers.
- Norway as a leading country in cancer prevention—This includes continuing preventive measures related to tobacco, nutrition, physical activity, and alcohol. The goal concerns both population measures to prevent the development of habits that increase the risk of cancer, and prevention in all parts of health and care services to change unhealthy behaviors and thus prevent the development of cancer.
- Increasing the number of people who survive cancer and live longer—Norway’s goal was to be among the countries with the highest 5-year cancer survival rates and lowest mortality rates. This goal included establishing quality registries for cancer care and developing new QIs.
- Ensuring the best possible quality of life for cancer patients and their families—Establishing treatment, rehabilitation, and follow-up plans adapted to the growing number of cancer patients [114].

The third national strategy, “Life with Cancer” (Leve med kreft), was developed for the years 2018–2022 (extended until early 2024) and was to lay the foundation for further improvement of high-quality Norwegian oncological care. This strategy was both a continuation and an update of the National Cancer Strategy 2013–2017—“Together Against Cancer”. The most important achievement of the previous strategy was the introduction of 28 cancer treatment paths. These paths provided patients with greater predictability of diagnosis and treatment, allowing procedures to be carried out as quickly as necessary. Patients confirmed that this was of great importance in terms of their sense of security. The third edition of the strategy focused on the same five goals that were adopted in the strategy for 2013–2017, but they were updated and expanded with additional activities [115].

The latest cancer strategy covers the years 2024–2028 and may also be extended for the following years. The current strategy continues to be based on the same five main objectives as in previous strategies. In addition, the new cancer strategy is in line with European plans and investments in the field of oncology. The main themes of European investments are:

- Reducing the number of cancer cases;
- Increasing the number of people surviving cancer;
- Improving the quality of life for people affected by cancer.

In 2021, the European Commission launched a comprehensive initiative in the field of oncology, “Europe’s Beating Cancer Plan” and “E.U. Mission Cancer”. These initiatives are funded respectively by the European health program EU4Health and Horizon Europe, which is the European research and innovation program. Norway, like the EU Member States, participates in both programs [116]. The main goals of the strategy for 2024–2028 are presented in Table 9.

An analysis of the quality of cancer care would not be complete without mention of the Norwegian registries. The Norwegian Cancer Registry was established in 1952, second only to the Danish registry. It collects detailed information on cancer treatment in nine quality registries. One of the main purposes of these registries is to provide data on whether cancer treatment is carried out in accordance with national guidelines. Quality registries of this kind with extended data also exist in other Nordic countries but are not monitored by the national cancer registry [98]. The Norwegian Cancer Registry, together with registries from these other countries, participates in the NORDCAN project. NORDCAN is a comprehensive database of cancer statistics for the Nordic countries: Denmark, Finland, Iceland, Norway, Sweden, the Faroe Islands, and Greenland. It contains data on cancer incidence, mortality, morbidity, and survival rates for more than 50 types of cancer. The data in NORDCAN are updated regularly and are more up to date than many other global databases. Data are provided by the Association of Nordic Cancer Registries and come from national cancer registries and death registries [117].

The actions initiated by the implemented cancer strategies have contributed to improved survival rates among Norwegian patients. In the case of all the most common cancers (colon cancer, rectal cancer, lung cancer, melanoma of the skin, kidney cancer, breast cancer, uterine cancer, ovarian cancer, and prostate cancer) over the years 1990–2015, significant improvements were observed in both 1- and 5-year relative survival rates [118].

### 3.13. Israel

Despite the fact that Israel currently does not have a national cancer plan [119], it is mentioned in many studies on QIs as an example of a country where systematic assessment of the quality of the health system using indicators has been carried out for years [120,121,122]. Furthermore, for the first time, a Country Cooperation Strategy (CCS) for 2019–2025 has been developed in partnership between the WHO and the Government of the State of Israel. The CCS is a medium-term framework for strategic cooperation between the partners, which outlines a joint program with priority areas of work for six years. The CCS includes four strategic priorities, which are presented in Table 10.

Within priority 2, the area of interest includes, among others, “Solving the problems related to non-communicable diseases as the leading cause of death, in particular, circulatory system diseases, respiratory system diseases, cancers and diabetes in relation to risk factors such as tobacco and alcohol consumption, nutrition and physical activity”, and the proposed goal for this area specified in the document is to reduce premature mortality due to circulatory system diseases, cancers, diabetes, and chronic respiratory diseases by 25% by 2025 [123].

In March 2004, the Israel National Institute for Health Policy and Health Services Research, together with the Health Council, launched the National Program for Quality Indicators in Community Healthcare in Israel (QICH). The program began in 1999. Many of the QICH indicators are based on definitions from existing international measures, such as those included in the Healthcare Effectiveness Data and Information Set of the National Committee for Quality Assurance in the United States, with the intention of conducting international comparisons. Since the establishment of the QICH, four national reports on the quality of healthcare in Israel have been published [124]. The first program report was published in 2004 and presented data for the years 2001–2003, with the remaining reports presenting data for subsequent years. In May 2010, the administration of the program changed, and its implementation was entrusted to a team from the School of Public Health of the Hebrew University of Jerusalem and Hadassah. One of the measurement areas included in the program is the indicators of screening tests for early detection of cancer. They currently include five indicators:

- For cervical cancer:
  ○ Performing a screening test to detect cervical cancer;
  ○ Failure to undergo cervical cancer screening.
- For colon cancer:
  ○ Screening for early detection of colon cancer;
  ○ Performing a colonoscopy after a positive occult blood test result.
- For breast cancer:
  ○ Performing a mammogram [125].

The QICH collects and shares information about how the Israeli healthcare system works. This allows both decision-makers and patients to see what is working well and which areas need improvement. The QIs used are based on the latest research and are in line with global standards. They are clearly defined and easy to measure, and selection of them is carried out in cooperation with various organizations, including insurers. The data are collected from all patients in Israel and are publicly available [124].

After Israel officially joined the OECD in 2010, the OECD prepared a report on medicine in Israel (published in October 2012). After a visit by representatives of the organization, the QICH program mentioned in the previous paragraph received positive recognition. However, room for further improvement in hospitals in Israel was recognized. The burden on hospitals and disparities in health indicators among the population were criticized. Among other things, the OECD recommended that hospitals establish a system for monitoring QIs and publish the results of measurement publicly. At the same time, accreditation programs should be developed and quality standards within organizations improved. The legal basis for monitoring QIs was established in 2012, with the adoption of the national health insurance regulations. Based on these regulations, the National Quality Indicator Program in Hospitals was launched, supported by an advisory committee for the definition of QIs and professional committees in all areas of medicine subject to measurement. The national program was launched as an expanding program in 2013. Significant clinical achievements were achieved during the initial years of its implementation. In the first year, five clinical QIs were measured in all general hospitals in the country, and the improvement in these indicators was visible in the first year of measurement. At the same time, five additional indicators were formulated for the beginning of measurement in 2014, geriatric and psychiatric hospitals joined the program at the beginning of 2014, and in 2015 the program was expanded to include other areas of medicine, such as breastfeeding, pre-hospital ambulance service, and psychiatric services [126].

The main objective of the National Quality Indicators Program for General and Geriatric Hospitals, Psychiatric Hospitals, Mother and Baby Health Centers, and Emergency Medical Services (Ambulances) is to promote high-quality healthcare in selected key areas of the Israeli healthcare system through a process of measuring the quality of care and making the results public [127,128,129,130]. One of the indicators monitored by the program is the indicator for appropriate antibiotic treatment in the case of surgery—colon and rectal surgery. These indicators have improved from the first year of measurement to the last year—from 78% in 2016 to 95% in 2020. From 2022, the polyp detection rate (PDR) in colonoscopy [127] has been added to the Israeli quality program. The detection and removal of polyps is a significant factor contributing to the decrease in the incidence of colon cancer. In 2022, the national detection rate was 37%, which is lower than the rates described in the literature (approx. 43%), but considering that this is a new indicator, the authors of the report expect improvement in the future [128].

The Israel National Cancer Registry (INCR) was established in 1960, and reporting of newly diagnosed cancer cases has been mandatory since 1982. The registry collects data on all malignant tumors (excluding basal cell and squamous cell carcinomas of the skin), carcinoma in situ and high-grade intraepithelial neoplasia, and benign tumors of the brain and central nervous system. The sources of information for the INCR are hospitals, pathology and cytology laboratories, and death certificates from local health authorities. Demographic data are obtained from the Israel National Population Registry and include name, date of birth and death, sex, health status, ethnicity, and address. In each case, trained registrars use available records to determine the location and morphology according to ICD-O-3 coding and to record the grade, size, treatment, and stage of the tumor according to the SEER Staging Manual. The INCR covers the entire population of Israel (approximately 8 million). The completeness of INCR data for solid tumors is estimated to be 95–96%. Data quality control activities are conducted by the Israel Center for Disease Control. INCR data are used by government ministries, researchers, and the public for a variety of purposes, including tracking cancer incidence and survival, formulating health policy, assessing the effectiveness of screening and treatment programs, and conducting research and health promotion activities [131].

Data from Israel show a positive impact of introducing QIs for breast and colon cancer screening. Weisband, Torres, Paltiel, et al. [132] examined differences in screening trends following the introduction of QIs. The study analyzed data on cancer screening among Israeli women between 2002 and 2017. It explored the frequency of screening and whether this was associated with the socioeconomic status and education of the participants. It was found that the most common screening test was for breast cancer, followed by for colon cancer and cervical cancer. Interestingly, women with higher socioeconomic status were more likely to be screened for cervical cancer than women with lower incomes. Trends over time showed that breast and colon screening rates increased after the introduction of QIs in 2004 and 2005, respectively, and that the greatest reduction in differences between the two occurred for breast cancer screening. The cervical cancer screening QI was introduced in 2015 and has not changed significantly to date. The study demonstrated increased uptake and reduced socioeconomic disparities after the introduction of cancer screening indicators. It was hypothesized that the cervical cancer screening rate would also translate into increased participation and a reduction in the socioeconomic disparities observed, as it had for breast and colon screening following the introduction of QIs. Findings from the Israeli Quality Indicators Program underscore the importance of primary care physicians as a driving force in increasing cancer screening rates to improve outcomes and reduce disparities.

### 3.14. Japan

In Japan, cancer control efforts began in 1983 with the introduction of the “Comprehensive 10-Year Strategy for Cancer Control” (1984–1993) as a national cancer control program. After that period, the “New 10-Year Strategy to Overcome Cancer (1994–2003)” was launched, with the emphasis in the first 20-year period being mainly on basic research. Then, in 2004, the “Third-Term Comprehensive 10-Year Strategy for Cancer Control” (2004–2013) was introduced to further promote cancer research and provide high-quality medical services to cancer patients. The main goal, as stated in the strategy, was to “drastically reduce cancer morbidity and mortality”, for which purpose increasing levels of funding were to be provided. The third strategy was not only aimed at funding cancer research but also at introducing activities for preventing cancer, improving the quality of cancer care, promoting social support systems, and establishing more effective systems for monitoring cancer incidence and survival. The organization with responsibility for managing the program was the Japan Foundation for the Promotion of Cancer Research [133].

**Figure 6:**
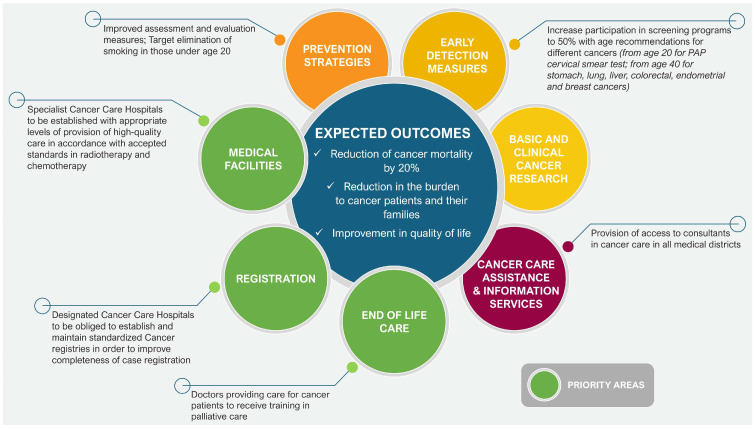
Basic Plan for Promotion of Cancer Control Programs (Phase 1) of the Japanese Ministry of Health, Labor and Welfare. Source: Cancer Research and Control Activities in Japan—Contributions to International Efforts [133].

The Cancer Control Act, passed in 2006 and implemented in April 2007, included three core areas of activity: prevention and early detection, reducing inequalities in care, and promotion of research. An important feature of the Act was that it allowed patient advocacy groups and other stakeholders to participate formally as members of the Cancer Control Promotion Council in collaboration with the Minister. The cancer registry was also acknowledged for its critical role at the center of cancer control activities. The Basic Plan to Promote Cancer Control Programs was introduced in 2007 (Phase 1) and covered the years 2007–2011. The main objectives were to reduce cancer mortality, reduce the burden on patients and their families, and improve quality of life. The plan covered seven specific areas, with three areas of particular interest: chemotherapy and radiotherapy, palliative care in the early phase of treatment, and promotion of cancer registration, which are highlighted in Figure 6 [133].

The next Basic Plan was initiated in 2012 as phase 2 of Japan’s cancer control plan, with the aim of ensuring that quality of care was a major goal of cancer control policy. The previously mentioned Cancer Control Act of 2006 required the Japanese Government to maintain the quality of cancer care across the country. Cancer hospitals were established nationwide (397 hospitals as of April 2012) to function as centers for providing high-quality care. Despite meeting the structural requirements of the plan, such as ensuring the availability of radiotherapy equipment and palliative care teams, the impact on actual patient care and outcomes was unclear. To address this, the Japanese Society of Clinical Oncology published 5-year patient survival rates from its member institutions, specifying the expected rigorous quality standards, which included provision of patient follow-up procedures. The intention was that the established network of cancer hospitals should follow the set criteria and procedures to ensure a national system for monitoring outcomes. Expert collaboration led to the development of 206 QIs for five common cancers and palliative care. These indicators, specifying target populations and care standards, were implemented by the Cancer Registry Chapter in 2012 [134].

In 2013, a year after the second phase of the plan was implemented, the Cancer Registration Promotion Act came into effect, which established the National Cancer Registry and required all hospitals in Japan to send basic information about newly diagnosed cancer patients to their territorial prefectures. This led to the foundation of the National Cancer Registry System (NCRS), an online system that collected information from 47 prefectures. It was launched in 2016, and in 2017, national incidence rates were first reported to the Ministry of Health, Labor and Welfare. In addition, the National Cancer Registry set up a database (the Population-Based Cancer Registry Database System—PBCRDS), which made it possible for prefectures to enter data from previous years into the cancer registry, and this was then merged with the current data in the NCRS. As of March 2020, forty-five registries had implemented the PBCRDS. Care has been taken to ensure that the National Cancer Registry conforms to the highest professional standards by conducting an external audit with regard to the security of the information secured at registration and a simplified registration system that requires only the submission of dates. The “Hos-CanR Lite” is available to hospitals [135].

In line with maintaining policies for the comprehensive and planned control of cancer, in 2014, the 10-year Strategy for Cancer Control was initiated, and 4 years later in 2018, the next stage of the Basic Plan to Promote Cancer Control Programs (Phase 3) was launched. The third phase of the plan was based on three main goals [136]:

1st  Evidence-based prevention and screening;
2nd  Patient-centered cancer treatment;
3rd  Creating a society in which citizens may live with dignity and safety.

In the 2018, Basic Plan support was announced for genomic medicine in cancer treatment, which in Japan was lagging somewhat behind Europe and the USA. A system of insurance was established, allowing citizens to access gene tests, and the data obtained on genomic mutations provided support for research into new drugs and early diagnostic methods, as well as the development of human resources in the field of genetic counseling. In addition, a law on clinical trials was also introduced, together with guidelines for funding research on as-yet-unapproved pharmaceutical products for potential treatment. Approval was also obtained for the introduction of a new vaccine for the control of cervical cancer in Japan [137].

As previously mentioned, QIs can be used as a tool to verify adherence to clinical practice guidelines. The main objective of a study conducted by Watanabe, Mikami, Katabuchi, et al. [138] was to propose a set of QIs based on clinical guidelines for the treatment of cervical cancer published by the Japanese Society of Gynecological Oncology and to assess adherence to the standards of care. A total of 297 hospitals participated in the study, and the care of 15,163 patients with cervical cancer was assessed, with the aid of 10 measurable QIs. The lowest adherence rate, of 50.0%, was reported for the procedure of “cystoscope or proctoscope for stage IVA”, and the highest, of 98.8%, for “chemotherapy using platinum for stage IVB”. Thus, although considerable variations in care were indeed found, hospitals reported clinically valid reasons for more than half of the cases of non-adherence. These accounted for 75%, 90.9%, 73.4%, 44.5%, and 88.1% of non-adherence. This variation in care patterns is suggestive of differences in compliance with standards of care across Japan, although the authors are careful not to dismiss them as signs of poor management. On the contrary, they point out that while the proposed standards of care may be valid in so far as they have been evaluated in appropriately designed randomly controlled trials, this does not necessarily translate into more complex, everyday clinical settings, where many patients have comorbidities and multiple primary cancers. They suggest that further evaluation of the causes of variation and non-adherence is needed to identify areas for potential improvement in patient care.

Another example of the use of QIs in the verification of adherence to clinical guidelines in cancer treatment is a study by Iwamoto, Nakamura, and Higashi [139], in which the authors assessed 13 quality-of-care process indicators using a claims database linked to a registry. The patient sample consisted of 128,353 cases, representing 94.2% of those for whom data were available and who had been diagnosed in 2011 with breast, prostate, colorectal, stomach, lung, liver, or cervical cancers at 178 designated cancer care hospitals participating in the study. Two of the QIs examined related to pretreatment testing, nine to adherence to clinical treatment guidelines, and a further two to supportive care for patients. Pretreatment testing was found to be in keeping with QIs at high levels (above 80%) in most hospitals, but for adjuvant radiotherapy and chemoradiotherapy compliance was low (20–37%), with the exception of breast cancer (74%). Conformity to QIs for adjuvant chemotherapy and supportive care varied considerably across hospitals (45–68%). Non-adherence to care standards in accordance with clinical guidelines was reported in 26 hospitals, where in the case of adjuvant chemotherapy, it was found that there were clinically sound reasons for not offering this specific form of care to the majority of patients who had not received this recommendation (greater than 70%). However, there appeared to be insufficient reasons for the failure to use adjuvant radiotherapy (52–69%) and a lack of provision of supportive care (greater than 80%). The authors suggested that the reasons for non-adherence to clinical guidelines should be further assessed in the future to improve the delivery of cancer care.

### 3.15. Australia

In Australia, cancer control has been recognized among five National Health Priority Areas (NHPAs) in response to the increasing health needs of the population and the increasing burden on the health system resulting from the occurrence of many chronic diseases, including cancers (mainly lung, skin, cervical, breast, colon, and prostate cancers). The NHPA Plan was developed and initiated in 1996 (release date 1998) and represents a key initiative for improving clinical outcomes, including mortality and morbidity among cancer patients. Most importantly, it provided recognition of the fact that for interventions to be effective in reducing the burden of illness, they needed to adopt an integrated approach to care, providing an evidence-based continuum from prevention, through treatment, to appropriate management and maintenance [140]. Through these initiatives, the Australian Government, with the support of the National Health and Medical Research Council, set out to prioritize areas for health, which led to the first reports outlining key issues and strategies in a number of areas. The plan built on earlier international initiatives, such as the WHO’s “Global Strategy for Health for All by the Year 2000” [141], and on national health documents, including “Health for All Australians” in 1988 [142], which defined goals and guidelines to reduce health inequalities and improve the overall health of Australians. The first report produced by the National Health Priorities Committee was the result of a collaboration with the National Cancer Control Initiative (NCCI), which provided expert advice regarding cancer control, and from the Australian Institute of Health and Welfare (AIHW), which undertook statistical analysis of cancer data. The NCCI was established in February 1997 and tasked with the development of a comprehensive national cancer control plan, and with the responsibility for providing the Health Ministry with independent, expert advice on all matters relating to cancer control in Australia. An important contribution from the NCCI was a report published in July 1997 entitled “Priority Issues Discussion Paper on Cancer”, which provided detailed information on the incidence, mortality, and estimated costs of cancer in Australia. This paper also described the priority-setting process that the NCCI had undertaken as part of the National Cancer Control Plan and Implementation Strategy, submitted to the Government in December 1997.

The NHPA Cancer Control Report identified a number of actions that might help to improve the situation for cancer patients. These included strengthening the decision-making role of patients in both curative and palliative care; increasing the availability and effectiveness of prevention programs, screening tests and treatment, rehabilitation, and supportive and palliative care; reinforcing the relationship between research and decision-making in cancer prevention and treatment; promoting evidence-based practice; improving and maximizing the use of data as a basic tool in decision-making; and encouraging the development of model centers of excellence in cancer care [140].

However, over time it has become clear that the earlier programs, although useful, were too general. Additionally, they lacked reporting indicators and detailed implementation strategies. Therefore, the initiative launched in 1996 became more focused and systematic (NPHA reports are produced every two years) and aimed at analyzing the impact and effectiveness of the actions taken and better understanding and management of the health problems of the population. In subsequent years, cancer control policy in Australia developed towards a more integrated approach to care, particularly towards prevention and screening. One example is the “Priorities for Action in Cancer Control 2001–2003” (PACC), launched in 2001, where the focus was on prioritizing actions in terms of cost-effectiveness and potential impact on the health of the population [143]. The priority actions covered the following areas: prevention, screening and early detection, treatment, and supportive and palliative care. Of the 13 priority actions, seven were included in the program “Budgeting and Marginal Analysis”, and all were assessed as being cost-effective. Of the eight cancers (non-Hodgkin’s lymphoma was added in 1998 as the eighth) listed by the NHPA as being priority cancers, seven were included in the priority actions. The priority actions serve to augment other cancer control efforts at a national level, including those in the NHPA initiative and the NCCI plan, and it was deemed appropriate that progress regarding their implementation should be reviewed every three to five years. Although the PACC report did not include a plan to implement the priority actions, it recommended that one should be developed in consultation with key stakeholders [143].

Currently, the main initiative in cancer care is Cancer Australia, established by the Australian Government in 2006 as the national advisory and coordinating body for cancer prevention, early detection, treatment, care, and research. Cancer Australia aims to reduce the impact of cancer, address disparities, and improve outcomes for people affected by cancer by leading and coordinating national, evidence-based interventions across all stages of care. The main assumptions and goals of the plan are presented in Table 11.

**Figure 7:**
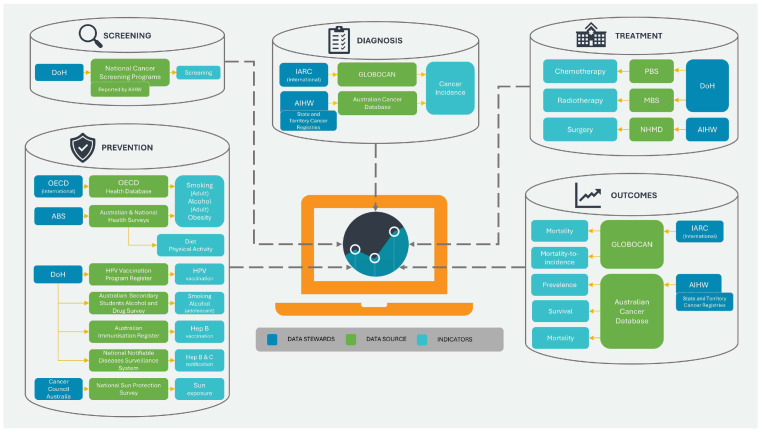
Source: NCCI data (reprinted with permission from Ref. [146]. Copyright 2024—Cancer Australia).

As part of its efforts to improve the quality of cancer care, Cancer Australia launched the National Cancer Control Indicators (NCCI) website as a dynamic interface that aggregates national data to provide information as to where efforts to improve quality of care can best be directed [144]. The NCCI includes a set of indicators that address the entire cancer care process, from prevention and screening to diagnosis, treatment, psychosocial care, research, and outcomes. The NCCI enables users to see the interconnections and relationships across the entire spectrum of cancer control activities, as well as to monitor national trends and conduct international comparative analyses. The data presented on the website are intended for a wide audience, including policymakers, governments, scientific societies, researchers, healthcare providers, and consumers. These data serve to increase understanding of the current situation, stimulate research, and inform future directions for cancer control, whether in research, policy, or clinical care. NCCI users can decide for themselves what type and level of information they wish to consult, as a result of interactive graphs that provide a visual representation of each indicator [145]. Figure 7 shows how the NCCI combines different data sources, including five domains, as the remaining two sections of the site are still under development [146].

From 2021, the main activity dedicated to improving the situation of cancer patients is the Australian Cancer Plan. Development of the plan was initiated by the Australian Government with support from Cancer Australia, and included objectives for prevention, early diagnosis, treatment, and palliative care for cancer patients. The development process involved extensive consultation with representatives from the different states, First Nations communities, patient advocacy organizations, researchers, and health professionals [144]. Among the observed changes, it is possible to extract information on the five-year survival rate from diagnosis for the periods 1982–1986 and 1992–1997, showing that it increased from 44% to 57% for men and from 55% to 63% for women, though this improvement cannot be attributed to specific initiatives [147]. More recent data indicate that the survival rate has increased to 71% for all cancers, which may be due to improved diagnostics and prevention programs. The impact of prevention programs is also reflected in the number of cancer cases detected, which has doubled from 88,000 in 2000 to around 169,000 predicted cases in 2024, although according to the AIHW, this increase is attributable in the main to increases in population size and an ageing population in whom cancer rates are higher. Furthermore, the age-standardized incidence rate of cancer has fallen from a peak of 508 cases per 100,000 of the population in 2008 to an estimated 486 cases per 100,000 of the population in 2021. This appears to have been driven primarily by significant declines in the incidence of prostate cancer (the AIHW indicates that this is probably related to earlier changes in diagnostic guidelines.) With the exception of prostate cancer, the incidence rate for all cancers in men has been relatively stable over the past 20 years. On the other hand, the incidence rate for all cancers in women has increased from 404 to an estimated 441 cases per 100,000 women. The rising incidence rates in women reflect, among other things, the increase in the rate of lung cancer due to the historical increase in smoking rates among women. Over the past 20 years, the incidence rates for most common cancers have increased, but for some cancers they have remained stable, and cancer mortality rates have continued to decline (with the declines more pronounced in men than in women). Between 1989 and 2021, age-standardized cancer mortality rates declined substantially in both men and women, from 287 to 182 deaths per 100,000 men and from 165 to 122 deaths per 100,000 women [148].

In Australia, the tool used to collect information on cancer in the healthcare system is the Australian Cancer Database (ACD). The ACD, as a national collection of information on cancer patients, contains data collected since 1982 on all cancer cases (excluding primary and squamous cell skin cancers). There is an obligation for designated personnel and organizations to provide data, which results from the applicable legal regulations. The institution responsible for the standardization and appropriate preparation of data, ensuring their completeness and reliability, is the AIHW. Thus, the ACD contains data at both the patient and the specific cancer level, including information such as date of diagnosis, age at diagnosis, topographic and histological codes according to ICD-O-3, behavior code, and stage at diagnosis. The ACD is the primary source of information for monitoring trends in cancer incidence in Australia, supporting research and shaping health policy in the field of cancer prevention, diagnosis, and treatment [149,150].

## 4. Discussion

The growing burden of cancer, recognized by the WHO as one of the leading causes of premature death before the age of 70, together with the projected increase in the incidence of cancer, clearly indicates the need for both monitoring and appropriate standardization of the process of assessing the quality of care. Monitoring is considered a tool that can support the development of medical standards and significantly contribute to the implementation of high-quality care in accordance with these standards. Moreover, a properly implemented monitoring and evaluation system can support efforts to ensure access to healthcare services and reduce inequalities in both access and quality of services provided. Valid and reliable data are key elements of effective healthcare systems. Numerous national and global initiatives, such as the NQF in the USA, the Health Data Collaborative, and activities carried out by the OECD, IDB, and China Joint Study Partnership, aim to improve the quality of healthcare services and strengthen the patient’s voice, emphasizing the global interest in measuring the quality of healthcare. This translates into a clear need for coordinated and appropriate action, specific to a given area, to improve the quality of care, including monitoring this process [1,2,3,4,5,6].

**Figure 8:**
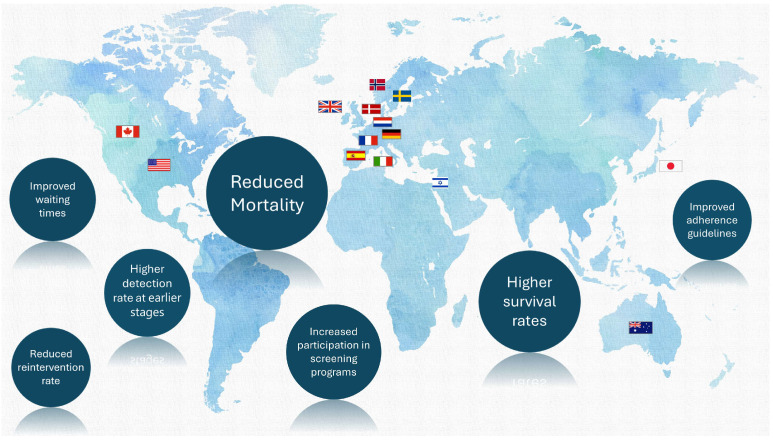
Most frequently indicated areas where improvement has been achieved.

As indicated in this review, the countries analyzed—the USA, Canada, Great Britain, the Netherlands, Germany, France, Italy, Spain, Sweden, Denmark, Norway, Israel, Japan, and Australia—despite there being significant differences resulting from different healthcare needs and priorities, have undertaken comprehensive actions to improve both the quality of life of patients struggling with cancer and to reduce cancer mortality. The initiatives implemented, although different in scope, intensity, dynamics, and implementation strategies, show quite similar and common, but to some extent unique, approaches in the field of prevention, early diagnosis, treatment, and care of cancer patients, which, in the long term, are expected to have a positive impact, expressed in increased detection of cancers at earlier stages and reduced mortality. All countries included in the review have, to a greater or lesser extent, achieved improvement in the outcomes for cancer patients (from prevention and screening programs, through diagnosis, to treatment and palliative care). The most frequently mentioned area of improvement was a reduction in cancer mortality rates and an increase in survival rates at 5, 3, and 1 year(s). Additionally, many countries have reported an improvement in adherence to guidelines as well as a reduction in waiting times for services. Figure 8 illustrates the most frequent achievements in improving the quality of cancer care. Countries such as the United States, the United Kingdom, Canada, the Netherlands, Australia, and Israel can be considered pioneers in implementing QIs into their healthcare systems, which has resulted in significant improvements in cancer care in these countries. These indicators help assess adherence to clinical guidelines, timeliness of treatment, safety of practices, and overall patient survival. The Scandinavian countries, on the other hand, can serve as role models for implementing IT tools, including registries that allow for ongoing monitoring of developments in healthcare.

The use of QIs as a tool in the process of assessing the quality of care for cancer patients is of great importance at every stage, from prevention, through diagnosis and treatment, to palliative care. As precisely defined measures, they are used to assess the effectiveness, efficiency, and safety of medical services provided as part of cancer care. They are an invaluable tool in the continuous process of improving the quality of care for cancer patients. Most highly developed countries systematically assess quality using these indicators, and the best basis for their development are clinical practice guidelines, because they allow for the identification of key points that require monitoring and have the greatest potential for improving patient outcomes. The usefulness of QIs based on clinical guidelines is much higher than, for example, the 5-year survival rate. Despite different opinions on the usefulness of the 5-year survival rate as a measure of success in the fight against cancer, in almost all national studies, it is used to present the positive impact of implemented actions on improving the outcomes for cancer patients. In a study conducted by Li, Pan, Kashaf, et al. [151], the authors discuss the limitations of using 5-year survival rates as a measure of progress in cancer control. While 5-year survival rates are commonly used to assess the effectiveness of treatment for cancer patients, the authors argue that they have several important limitations. Among these is the contention that limiting the monitoring of progress in cancer treatment and control only to observed improvements in 5-year survival rates may be somewhat short-sighted. They point out that improvements in this indicator may be due to many factors, such as improved living conditions of the population (better health, employment, education), advances in oncology that translate into increased effectiveness of therapy and thus extend the lives of cancer patients, and early detection and screening (early diagnosis without a change in the course of the disease is associated with the fact that patients are monitored at earlier stages in the disease process and therefore appear to live longer after diagnosis). In this context, the authors believe that 5-year survival may not be evidence of progress in efforts to control cancer or even of any improvement in clinical treatment, perhaps demonstrating instead improvements in diagnosis. In another study of the validity of using the increase in 5-year survival as a measure of success in cancer control, by Maruvka, Tang, and Michor [152], the authors proposed a new approach to assessing the effectiveness of clinical interventions using information based on the incidence, survival, and mortality of cancer patients. They attempted to control for the alleged lead-time bias in 5-year survival rates by making a comparison with mortality normalized by incidence (MOI) and found a strong pattern of correlations between the two for all the countries examined in their study. This suggests that early diagnosis is not a major problem for using changes in survival rates to analyze data. The authors conclude that the increases in 5-year survival rates reflect real improvements over time in clinical cancer care. They propose that using a joint measure of survival with MOI would be optimal in measuring the degree of improvement in cancer treatment, especially because it is rare for one measure to be a perfect indicator.

In addition to clinical guidelines, registries play an important role in the development and implementation of indicators—both national cancer registries and quality registries, which contain detailed clinical data. The vast majority of national cancer plans emphasize the key role of registries in the process of improving quality of care. It should also be noted that national plans or strategies, as well as initiatives undertaken by scientific societies, can be seen as the main driving force behind the development of QIs. Although the use of indicators is associated with certain challenges, such as the need for standardization and data availability, their importance for improving the quality of life of cancer patients is undeniable. The use of QIs in cancer care can help identify gaps and shortcomings in the diagnostic and therapeutic process. When assessments are made using indicators, the quality of care is reflected in the measures provided by the indicators; the more indicators that reach the expected values, the better the quality of care for patients. Some QIs achieve relatively low utilization rates in the patient population, probably because of the difficulties in implementing them in clinical practice. One example of this is the extent to which the availability of breast-conserving surgery is limited by a shortage of trained radiation oncologists. It has been suggested that interest should be focused on indicators that demonstrate wide variations in utilization across providers. Such variations suggest significant room for improvement and increase the likelihood that these will be key indicators with a large impact on quality of care [153].

Although this review focuses on narratives that allow for the presentation of outcomes in terms of implemented or ongoing strategies, the interest in measuring the quality of healthcare is also reflected in other work that represents a scientific and often critical approach to the issue of measuring quality using indicators. Indicators are not only a tool for assessment but also a tool for making informed decisions about resource allocation, developing health policy, and designing quality improvement programs. Their development and use require the involvement of many stakeholders.

One example representing a critical approach to the implementation of such solutions is the 2014 study by Spinks et al. [154], which assesses the American oncology care system, pointing out persistent quality gaps that have remained unresolved since the Institute of Medicine’s (IOM) report of 1999. The authors analyzed the main barriers to achieving high-quality care, emphasizing the importance of patient-centered care supported by effective quality measurement systems. One of the key problems was the lack of systemic measurement tools, which made it difficult to implement the IOM recommendations. The significant role of the patient–doctor relationship was also indicated, which can affect treatment outcomes. An important postulate of the study authors was to pay special attention to the creation and development of the Learning Health System, which, thanks to real-time data analysis, would enable better decisions to be made and thus improve treatment outcomes. In addition, the authors noted the difficulties in implementing initiatives for oncological care as a significant problem, especially in the context of quality measures that do not take into account the needs of patients and the lack of validated indicators specific to oncology. According to the authors, the integration of quality measures and the treatment of quality measures as a fundamental tool should be considered an important factor supporting progress in oncological care.

Efforts to evaluate the implementation of QIs have also been the subject of other reviews. A study by De Vos et al. [155] examined the effectiveness of implementing QIs in hospital care and their impact on improving the quality of care. The systematic review covered the literature from 1994 to 2008,in particular, studies on interventions based on QIs such as audit and feedback. Twenty-one studies were included in the review, although mainly from the area of cardiac care. The results of the study suggest that implementing QIs as a tool for improving hospital care requires a comprehensive approach that combines different implementation strategies. The study showed that the effectiveness of implementation strategies depends on their structure, as those that combined feedback with education and quality improvement planning were the most effective. As the authors pointed out, these results are consistent with previous studies that confirm the effectiveness of multi-faceted interventions in improving the quality of healthcare, such as that of Foy et al. in 2005 or the later Ivers et al. study from 2015 [156,157]. Multifaceted approach implementation strategies allow for more comprehensive support for medical personnel, which increases the chances of achieving lasting changes in clinical practices. However, it should be noted that such analyses, despite the results achieved and the possibility of formulating constructive conclusions based on them, also reflected in other studies, are highly susceptible to the possibility of generalizing conclusions, primarily due to the lack of detailed information among all the studies included in the review or the narrow scope of the clinical areas analyzed.

In HICs, data-driven quality measurement programs began in the 1990s [22]. Thus, these countries now have extensive experience with quality assessment and are able to clearly articulate areas of success and failure. Given that HICs have seen improvements in the quality of cancer care provided to patients, the next challenge has been to develop QIs in low- and middle-income countries (LMICs). To meet expectations for improving quality of care, ASCO, in collaboration with representatives from lower-resource countries, developed a pilot QOPI pathway with the goal of making quality improvement assessment more accessible and adaptable to the changing conditions in LMICs. An important aspect was ASCO’s collaboration with oncologists and cancer centers in LMICs to adapt standardized QOPI measures. In the QOPI LMIC pilot, the first step toward quality improvement was learning how to collect and report data. Identified data are extracted and submitted via online platforms to provide comparative results on identified quality measures. Data analysis is then conducted and quality improvement steps are initiated. Two rounds of reporting per year provide reports on outcomes for individuals or practices [158]. Data-driven quality improvement and assurance initiatives using validated indicators can add value to cancer health systems in low- and middle-income countries. In addition to the obvious focus on improving quality of care, measuring, reporting, and acting on QIs build verifiable confidence in the health system and those who govern it. For example, indicator results can be used to improve transparency, demonstrate that cancer care resources are achieving desired outcomes, or otherwise guide policy or funding changes [22].

Although the barriers to implementing pro-quality solutions in the selected countries were not the subject of analysis in this study, it might be possible to characterize them in terms of upcoming challenges. However, it should be emphasized that this would only serve as a spiritus movens for further research in this direction.

Future challenges related to QIs may include organizational issues such as coherence and consistency of the solutions implemented. Each strategy or plan is limited by a specific time horizon, and each solution has a specific number of objectives and actions defined that require implementation. As the number of programs and initiatives increases, the problem of fragmentation of activities arises, which can make it difficult to define priorities and assign clear responsibility for the results achieved. This has been highlighted in France, where previous cancer strategies were evaluated as being too short or too dispersed to achieve the intended goals [82]. Setting time limitations for the implementation of quality strategies though a critical factor in ensuring that progress is achieved within a foreseeable time frame may also prevent the appropriate division of competences and impede the search for the most optimal solutions. Another challenge resulting from technological progress, especially at a time of intensive development of artificial intelligence systems, lies in the problems related to the appropriate integration of data. The Scottish experience serves as an indication of the growing importance of digitalization. New tools for analyzing data on cancer therapies are being developed within the Scottish Cancer Network (SCN), which are then published as part of patient pathways on dedicated websites [57]. It may also be important to adapt QIs to the personalized care related to the more individualized type of healthcare services provided. Currently, for example, Canada, as part of the strategy implemented for 2019–2029, is focusing attention on the individual needs of patients and their preferences in the treatment process [40]. This approach may determine the need to change existing indicators or adapt them in order to obtain more reliable information on the quality of health services provided in a more personalized model of care.

Although the attempt to predict upcoming challenges in this study is only partial, some conclusions correspond with those of other studies addressing the issue of barriers in the implementation of quality improvement programs. In a study by Carbonell et al. 2024 [159], problems related to data collection and processing were indicated as one of the main barriers in the implementation of quality systems. The authors pointed out that issues related to privacy protection limited the possibility of monitoring and assessing the effects of implemented programs, which restricted the opportunities offered by digitization and the effective use of data. Furthermore, the study also identified other barriers to the implementation of pro-quality programs, including political, administrative, socio-economic, and educational factors. The authors also identified resistance to change and low staff involvement as resulting from the lack of clear financial incentives and limited time available for these activities. This probably means that medical staff and management often treat these initiatives as merely an additional burden. The aspects of financial support for such initiatives are also confirmed in the study by Pain et al. 2024 [160] which, in addition to barriers such as staff shortages, lack of sufficient time for pro-quality activities, and insufficient research infrastructure and data systems, identified them as the main problem in implementing quality systems. In another study by Hespe et al. 2018 [161] in Australian primary care, the authors indicated that, apart from the financial aspects and access to appropriate data and their effective use, additional challenges created an obstacle to the implementation of quality improvement initiatives. These concerned appropriate leadership and an organizational culture defined as offering active support through building local networks and strengthening teamwork, as well as supervisory organizations that should have a significant role in actively supporting the implementation of these initiatives. As regards political factors that may act as a barrier to the implementation of quality solutions, it should be noted that changes in government in individual countries, especially when the change results in a realignment of policy, often translate into changes in health policy, which may affect future priorities in healthcare.

In February 2025, the European Commission published a staff working document entitled “Review of Europe’s Beating Cancer Plan”, summarizing the activities undertaken within the European Cancer Plan, published in February 2021. A significant part of the document is devoted to the issue of the quality of healthcare as understood in the broadest sense. This is directly related to the main pillars of the European Cancer Plan, which include ensuring high standards of care and improving the quality of life of cancer patients, survivors, and caregivers. The authors of the document emphasize the importance of creating an EU Network of Comprehensive Cancer Centers to ensure high-quality multidisciplinary care, regardless of place of residence; building systems for monitoring and forecasting the burden of cancer in the EU, which would support decision-makers in adapting health policy to changing conditions; and the importance of using clinical practice guidelines to improve the quality of care. The report also emphasizes the importance of national cancer registries as a key source of data for identifying inequalities and measuring the effectiveness of healthcare systems. Unfortunately, despite the many activities carried out as part of the European Cancer Plan, the authors of the report point out a number of challenges or failures related to the implementation of strategies that EU countries are facing. Among them, they mainly mention financial barriers, related to the rising costs of healthcare or the insufficient number of medical personnel; clinical barriers, in particular, in the area of uneven application of clinical practice guidelines; political and institutional barriers, which refer to disparities in resources between regions and the lack of reliable data necessary to create evidence-based health policy; and cultural barriers, related to the lack of a common understanding of the challenges in the fight against cancer or the lack or insufficient interest on the part of healthcare workers. Finally, the authors emphasize that EU efforts should focus on prevention, which should consequently reduce the burden of disease or at least reduce its negative effects on society [162]. The conclusions drawn from the report, relevant to those presented in this review, confirm that intensified actions to improve quality of cancer care are still necessary.

Finally, it should be emphasized that this review, as well as many other studies focusing on the impact of quality monitoring and assessment, have their fundamental limitations. A major risk that may result from such studies is primarily the potential possibility of generalizing conclusions. First, not all analyses and reports provide detailed information on the implemented strategies and do not always include the presentation of detailed results that take into account differences in the characteristics of groups before and after the introduction of a given intervention, action or strategy, which may complicate a precise assessment of the effectiveness of specific initiatives. Second, it seems that the authors of the reports are reluctant to refer to unsuccessful initiatives and therefore do not present negative results, thus limiting the possibility of a critical look at the actions taken. Nevertheless, the main assumption is to identify the best possible solutions, which are an important driver for further work to improve quality. Certainly, the growing global burden of cancer requires a special focus on the quality of care. National healthcare systems still face many challenges related to improving the quality of oncological care, and limited resources for healthcare, an aging society, and lifestyle diseases should be an impetus for further development of and improvement in national systems for monitoring the quality of care. A comprehensive approach is needed to meet these challenges, encompassing both data collection, data analysis, and implementation of quality improvement initiatives. Thus, developing and using QIs suitable for this purpose would be expected to significantly improve the effectiveness of implementation and the quality of oncological care.

## 5. Conclusions

Quality indicators can be considered an essential tool used in the process of assessing the quality of cancer care. They help to assess the effectiveness, efficiency, and safety of treatment and have a direct impact on improving patient outcomes at all levels of care. Moreover, they are useful in identifying areas requiring corrective action, leading to continuous improvement in clinical practice. Valid, reliable, and rigorously controlled data are crucial for building effective systems for quality control of healthcare. Initiatives based on reliable data can translate into improved quality of cancer care. However, to effectively implement QIs, a comprehensive approach is required at all stages of the process, starting from data collection, analysis, and conclusions and then implementation of appropriate actions that translate into quality improvement. Multi-pronged strategies combining feedback reporting, education, and quality improvement planning can lead to lasting improvement of clinical practice. Additionally, the involvement of stakeholders from different fields of medicine and patient representatives are central to refining the process of developing and implementing QIs.

Despite many successes in improving the quality of cancer care, further systematic research is still needed to identify and validate new QIs that may be relevant to different healthcare settings involved in cancer care. International cooperation between countries with established experience in quality assessment and those that are still in the early stages of implementing quality assessment processes can accelerate the development of standardized approaches to quality measurement. As emphasized in all implemented cancer plans, the focus on patient-centered care should be a central element of all quality improvement efforts. Implementing effective quality improvement strategies based on scientific evidence can contribute to improving the quality of cancer care worldwide and thus improve patient outcomes, providing patients and their families with better quality of life both during and after treatment.

## 6. Limitations

It is not possible to conduct either a quantitative or a qualitative analysis based on the results of this review. The high heterogeneity, complexity, and specificity of healthcare systems in the selected countries significantly prevent direct comparative analysis. Additionally, although the study attempted to select countries with a highly developed quality monitoring system, some of them nonetheless have more advanced quality assessment systems, while others are in earlier stages of development, which significantly limits the possibilities for comparison. Another significant limitation is the lack of access to complete data and information concerning the time at which specific solutions were implemented. Data from individual countries are available from a variety of sources, often in highly heterogeneous forms without an overriding organizational framework, which necessitates the narrative form of presentation used to describe the benefits associated with the implementation of pro-quality initiatives. To achieve a more standardized comparison, it would be necessary to use an approach focusing only on specific endpoints for the specified solutions analyzed at a specific point in time. This, however, might result in somewhat limited or even erroneous conclusions, as the multifactorial nature of oncological care may not be captured adequately and lead to the incorrect interpretation of the cause-and-effect results. There is also a high risk of subjectivity in the results presented in the reports and studies included in the review. It cannot be ruled out that the authors of the studies used only “positive” results, which indicated the desired aspects of the implemented solutions. It should also be remembered that the study conducted covers only a fragment of the entire healthcare system within a specific time horizon of the analysis, which may not correspond to the actual situation or the results actually achieved by individual countries. Future studies are required to address this issue of the validity in implementing and maintaining quality assessment systems as a tool with a real impact on improving patients’ clinical outcomes and epidemiological indicators.

## Figures and Tables

**Table 1:** Summary of the three main priorities of the NHS England Long Term Plan.

Priority	Actions
Priority 1: Early detection	Enhancing public awareness of cancer Increasing emphasis on the role of primary healthcare, especially in terms of timeliness of referrals Using interventions focused on detecting cancer in patients at increased risk Further development of screening tests Using an approach based on predefined patient pathways Investment in new technologies supporting early detection Pipeline interventions to improve health outcomes in society
Priority 2: Improving medical treatment and personalized health services	Improving access to new technologies available in cancer diagnosis and treatment Reducing disparities in access and treatment between hospitals Improving services provided to children with cancer A patient-centric approach focused on improving the experience of care and quality of life of patients
Priority 3: Enhancing efficiency	Increasing investment in diagnostic infrastructure Providing support to trusts to better manage waiting times for services Further action to optimize diagnostic and therapeutic pathways

Source: NHS England Cancer Programme Progress Update—Spring 2024 [49].

**Table 2:** Summary of the 11 main areas of the Cancer Strategy for Scotland 2023–2033.

Ambition/Area	Action
Area 1: Prevention strategies	The main activities focus on reducing tobacco use, promoting healthy lifestyles, and increasing HPV vaccination rates in both girls and boys.
Area 2: Early detection	Increase detection in the early stages of cancer by improving early education and primary care support, and optimizing screening programs.
Area 3: Optimizing readiness for therapeutic intervention	Ensure that patients are well supported in the period between diagnosis and the actual start of treatment. This action involves prehabilitation in the form of education, promoting engagement in physical exercise, improving nutrition, providing the necessary psychological support, and addressing any underlying medical issues that might affect treatment outcomes.
Area 4: Comprehensive and efficacious therapeutic approach	Increase the integration of services across different specialist areas, providing a more comprehensive and systemic approach to care to ensure the best possible outcomes for patients.
Area 5: Post-treatment care and support	Provide aftercare that meets the individual needs of the patient, in terms of rehabilitation, general well-being, and post-treatment monitoring, as well as palliative care and care around death. Providing support to carers is also a priority.
Area 6: Optimize staff management	Create optimal employment conditions for medical staff, ensuring a safe working environment from the perspective of both healthcare providers and patients.
Area 7: Provide individualized care	A patient-centered approach ensuring that the patient’s personal dignity is respected, that the patient is involved in informed health decision-making, and that care is coordinated at all stages.
Area 8: Remove disparities	Ensure equal standards of care regardless of sociodemographic factors.
Area 9: Mental health support	Place greater emphasis on the availability of psychological care for both patients and their families. Include psychological care as part of primary health care.
Area 10: Basic and clinical cancer research	Improve access to clinical trials, and investment in molecular diagnostics and new technologies.
Area 11: Data-driven cancer information services	Invest in IT infrastructure to enable the collection, processing, and sharing of detailed cancer data, including the use of performance indicators as a key tool for quality improvement and optimization of care.

Source: The Scottish Government Cancer Strategy for Scotland 2023–2033 [55].

**Table 3:** Six flagship measures of the French National Cancer Plan for 2009–2013.

Area	Standard	Activities
Research	Measure 1: Increased funding for interdisciplinary research	− Accreditation of five integrated cancer research centers. These centers were to be selected through a competitive process and should contribute to faster transfer of research results into clinical practice. − Increase patient participation in clinical trials by 50%. Efforts should focus primarily on the most vulnerable patient groups: children, older people, patients with rare types of cancer, and severe forms of cancer.
	Measure 3: Identification of environmental and behavioral risk factors	− Devoting more than 15% of the plan’s research budget to the analysis of environmental and behavioral risk factors. − Contributing to the complete genome sequencing of the five most common cancers. This goal was part of the global effort to profile the cancer genome.
Observation	Measure 6: To prepare and share, on an annual basis, information about cancer, cancer research, and treatment	− Annual preparation of an analysis of the occurrence of cancer in the country.
Prevention—screening	Measure 14: Combating inequalities in access to and implementation of screening	− Increasing participation of the entire population in organized screening programs by 15%. − The level of increase should be 50% in areas experiencing greatest difficulties.
Patient care	Measure 18: Individualizing patient care and expanding the role of the referring physician	− Ensuring that 80% of patients had at least one individualized care plan. This plan should include involvement of the referring physician.
Life during and after cancer	Measure 25: Developing individualized psycho-social support during and after cancer treatment	− Ensuring that 50% of patients used at least one cancer care plan that addressed individual needs for medical supervision and psychological and social support.

Source: Cancer Plan 2009–2013, launched on 2 November 2009 in Marseille [78].

**Table 4:** Summary of the assumptions of the French National Cancer Plan for 2014–2019.

Area/Ambition	Goals
Curing more patients	Goal 1: Promoting early detection of diseases
	Goal 2: Ensuring the quality and safety of care
	Goal 3: Supporting technological and therapeutic development
	Goal 4: Developing oncology training and career pathways in oncological care
	Goal 5: Accelerating innovation for the benefit of patients
	Goal 6: Strengthening France’s leading role in personalized medicine
Preserving continuity and quality of life	Goal 7: Providing comprehensive, personalized care
	Goal 8: Reducing the risk of sequelae and secondary cancers
	Goal 9: Reducing the impact of cancer on personal life
Investing in prevention and research	Goal 10: Launching the National Nicotine Addiction Prevention and Treatment Program
	Goal 11: Ensuring everyone has the opportunity to reduce their risk of cancer
	Goal 12: Minimizing the risk of developing cancer caused by occupational or environmental factors
	Goal 13: Creating conditions for conducting innovative research
Optimizing management and organizations	Goal 14: Revitalizing health democracy
	Goal 15: Basing public policies on sound and publicly available evidence
	Goal 16: Optimizing the organization of care for greater efficiency
	Goal 17: Adapting financing systems to the needs of oncology

Source: Cancer Plan 2014–2019, launched on 4 February 2014 [80].

**Table 5:** Main priorities of the Italian National Oncology Plan 2023–2027.

Priority	Actions
Priority 1: Cancer Prevention	Strengthening the role of prevention as the most effective tool in the fight against cancer through actions aimed at eliminating risk factors (e.g., smoking, poor eating habits, lack of physical activity), screening tests and preventing disease recurrence.
Priority 2: Diagnostics and early detection of cancer	Development of screening programs for high-risk groups and improvement of access to diagnostics.
Priority 3: Oncology patient care	Increasing the role of comprehensive care for oncology patients (from diagnosis, through treatment, to rehabilitation and psychological support). The plan also includes improving equity in access to therapy and ensuring high quality treatment for all patients.
Priority 4: Integration and coordination of the oncology care system	Strengthening cooperation between hospitals, clinics and social institutions and establishing regional oncology networks ensuring continuity of care.
Priority 5: Research and innovation in oncology	Investments in innovative solutions aimed at improving treatment effectiveness.
Priority 6: Education and communication	Improving the quality of communication in doctor-patient relationships. Investments in training for medical personnel and information campaigns aimed at patients and society.

Source: The Ministero della Salute Piano Oncologico Nazionale: Documento Di Pianificazione e Indirizzo per La Prevenzione e Il Contrasto Del Cancro 2023–2027 [89].

**Table 6:** The main priorities of the Spanish National Health System Cancer Strategy.

Priority	Actions
Priority 1: Health promotion and prevention	Emphasis on promoting a healthy lifestyle and preventing cancer through eliminating risk factors and screening.
Priority 2: Cancer diagnostics and early detection	Improving patient access to diagnostic testing, including improving the scale and screening programs.
Priority 3: Oncology patient care	Increasing the role of comprehensive oncological care—from effective treatment to support in improving quality of life and palliative care. The plan assumes a change in the organization of treatment, including monitoring patients after therapy and rehabilitation, as well as the development of palliative care for patients in advanced stages of disease.
Priority 4: Cancer data	Investments in an efficient information system—cancer registries and outcome monitoring systems. The plan assumes the creation of a uniform system for collecting epidemiological data on cancers, enabling precise tracking of incidence, survival, and effectiveness of oncological care in individual regions of the country.
Priority 5: Oncological care for children and adolescents	Organizing specialized pediatric care on a nationwide scale by implementing national guidelines for the organization of cancer treatment in children and adolescents, so that care is coordinated, specialized, and equally available in all regions of the country.

Source: The Ministerio de Sanidad Estrategia en Cáncer del Sistema Nacional de Salud [92,94].

**Table 7:** Main areas and activities of the Swedish cancer strategy.

Area	Actions
The Public Perspective	
Prevention	Increase public awareness of the consequences of smoking (support for those wishing to quit, introduction of strict anti-tobacco policies) and overexposure to UV radiation (close cooperation between GPs and dermatologists, and introduction of age limits for sunbed users); action on cancer vaccine development.
Early detection	Continue to develop and increase accessibility to screening programs, bridging the gap between regions.
Provide knowledge to build public awareness	Development of services providing information on cancer to raise public awareness and provide knowledge on how lifestyle and eating habits can affect the development of cancer.
The Patient Perspective	
Communication and information	The main focus is on the provision of a multidisciplinary approach, individualized treatment plans, and dedicated contact persons within the diagnostic and therapeutic process.
Waiting times	Establish maximum waiting times for services and monitor them within functioning quality registers.
Follow-up	Assessment of quality of life and patient satisfaction should be monitored through national quality registers.
Management and development	Using data on waiting times, quality of life, and patient satisfaction in management information; appointing care coordinators; and involving patient carers in the oncology care planning process.
Children and young adults	Increased activities related to aftercare, long-term support, and the development of research related to the late effects of childhood cancer.
End-of-life care	Increased investment in the development of palliative care due to existing shortages and changes in society.
Availability and use of health technologies	Ensuring equal access to medicines and medical devices. Assessing the cost-effectiveness of marketed medicines and devices. Introducing evaluation standards for medical devices.
Provision of Knowledge and Experience	
Oncology centers and networking	Development of a network of regional cancer hospitals with the appropriate structure and resources to meet the requirements set out for comprehensive cancer centers over time.
Research	Increase investment in clinical research and innovation.
Improved monitoring of oncology care outcomes	Actions to collect, process, and use registry data more effectively, aiming for 100% coverage. Monitoring quality of care and monitoring medication use.
Ensure an optimal supply of human resources	Activate health authorities in the development of long-term basic and specialized training plans for the continuous improvement of medical staff.

Source: The National Cancer Strategy for the Future—Summary; Report of the Commission of Inquiry on A National Cancer Strategy; Stockholm 2009 [96].

**Table 8:** Summary of the areas targeted within Danish cancer plans.

Area	Actions
Prevention and early detection strategies	Each plan places emphasis on improving the prevention and early detection of cancer, with activities including both primary and secondary prevention, including increasing the coverage of and participation in screening tests.
Continuous training of medical personnel	Activities related to promoting continuing education and improving the skills of doctors, nurses, and other medical and non-medical staff.
First contact and referral for treatment	Educational activities and research on patient behavior in the context of late reporting to GPs in the event of the appearance of disease symptoms.
Diagnostics and examination	Striving for initial diagnostics to be conducted in primary care and extended diagnostics in hospitals.
Development of diagnostic and therapeutic infrastructure	Investment in diagnostic and therapeutic infrastructure. Ensuring adequate access to optimal diagnostics and therapy, including surgery, radiotherapy, chemotherapy, and systemic treatment.
Centralization of surgical treatment	Concentration of surgical treatment in specialized centers performing an appropriately high volume of operations.
Patient pathways	Patient paths introduced as part of the second edition of the plan are to ensure that all patients, regardless of location, receive the same standard of care. Paths provide patients with full transparency regarding the care process.
Basic and clinical cancer research	Development of clinical medical databases necessary for further development of scientific research.
Rehabilitation	Striving to provide individualized oncological rehabilitation to all patients who require it, in the shortest possible time.
End-of-life care	Investments in increasing access to palliative care facilities.
Patient-centered approach	Focus on the individual needs of the patient. Education in the field of self-care and building health awareness, along with the development of mechanisms of action in the event of deterioration of health, both physical and mental. This area also includes support for relatives and caregivers of the patient. The patient-centric approach is particularly emphasized in the third and fourth editions of the plan, especially with regard to improving the patient’s quality of life and experience at every stage of the disease.
Monitoring	Due to the high dispersion of cancer data, it is necessary to take action to increase reporting to existing registries and clinical databases, implement quality monitoring using indicators, and make the transition to electronic documentation.

Source: The four editions of the Danish National Cancer Plan [104,105,106,107].

**Table 9:** Main goals of the Norwegian National Cancer Strategy 2024–2028.

Goal	Actions
Norway as a leader in cancer prevention	By 2030, Norway aims to reduce premature deaths from diseases such as cancer by one third. This will involve reducing risk factors such as smoking, unhealthy diet, and exposure to the sun. Cancer screening is to be improved to detect diseases earlier. The intention is also to completely eliminate HPV-related cancers, and additionally to provide personalized prevention strategies in healthcare.
Norway as a leader in providing high-quality patient care	The strategy includes the establishment of comprehensive cancer centers (CCCs) across the country to provide high-quality, coordinated care for cancer patients. These centers are to integrate research and treatment, and speed up access to new treatments. It is assumed that patients will be able to transition seamlessly between hospital and community care.
More patient-centered oncology care	Patients are to have access to clear information and will be involved in their own care. Digital tools are to improve communication and patient engagement. There are plans to integrate home monitoring with treatment. Patient feedback is to be used to improve care. There is also an assumption of equal opportunities for participation in clinical trials.
Increase the number of people who survive cancer and live longer	Plans are in place to increase the number of cancer patients who will have access to personalized treatments based on their individual genetic profiles. The quality of care will be continuously measured and improved through the collection of data and feedback from patients. Patients will also be supported to manage long-term side effects and encouraged to continue working.
Providing the best possible quality of life for cancer patients and their families	It is assumed that new methods of treatment will minimize side effects. Rehabilitation and support in the event of long-term side effects are to be provided to as many patients as possible. Patients and families are also to receive support from patient organizations. It is planned to adapt palliative care to growing needs and ensure its availability throughout the treatment period and to train healthcare workers in palliative care

Source: The Nasjonal kreftstrategi 2024–2028 [116].

**Table 10:** Priorities of the strategy developed in cooperation between Israel and the WHO.

Priority	Actions
Priority 1—Involves using e-health innovations as a tool to improve access to and quality of health services	This includes developing priorities for public health actions to support the adoption of e-health; big data in health systems; ICD-11, International Classification of Health Interventions, and International Classification of Functioning, Disability and Health; digital medicine; health research; and innovation as tools to strengthen health systems and provide more advanced coverage to promote overall well-being.
Priority 2—Focuses primarily on improving health outcomes for patients across the lifespan	It focuses on non-communicable diseases and infectious diseases, with a particular emphasis on personalized medicine and genomics. Under this priority, the main objective is to reduce the burden of non-communicable diseases, such as cardiovascular diseases, cancers, and diabetes, and improve overall health by tailoring interventions to individuals.
Priority 3—Includes strengthening the preparedness of countries to prevent, respond to, and recover from various crises	This priority will provide support to improve crisis management systems, implement international health standards, and share resources such as ambulances and emergency medical teams.
Priority 4—Enhance Israel’s contribution to global health	This priority includes strengthening Israel’s role in global health by strengthening cooperation between the WHO and Israeli scientific institutions from various sectors.

Source: The Country Cooperation Strategy, Israel, 2019–2025 [123].

**Table 11:** Strategic objectives of the Australian Cancer Plan.

Strategic Objectives	Goals
Objective 1: Prevention and early detection strategies	Action aimed at creating a prevention and early detection system that will be based on scientific evidence and adapted to the individual needs of all citizens.
Objective 2: Improving the patient experience	Implementing system solutions focused on overall patient well-being. Providing an individualized approach to ensure that all patients have the best possible experience in contact with the healthcare system, which will translate into their outcomes and quality of life.
Objective 3: Providing high-quality patient care	Development of an advanced comprehensive healthcare network providing a coordinated approach to care. A healthcare system based on reliable data to optimize the patient path.
Objective 4: Data as the foundation for decision-making	Investments in new technologies that allow the use of data in the process of improving results. Defining common standards for data collection, adapted to the adopted priorities in the field of scientific research.
Objective 5: Ensure an optimal supply of human resources	Investment in staff to provide culturally safe care for all citizens. Continuous development and training in clinical safety to ensure the best quality of care.
Objective 6: Closing the gap in outcomes for different ethnic groups	Supporting equal access to high-quality cancer care across all ethnic groups. Preventing racism and discrimination.

Source: The Australian Cancer Plan; Australia 2023 [144].
